# Assembly of Protein‐DNA Framework Nanostructures: Structurally Defining Protein‐DNA Interfaces With Aptamer

**DOI:** 10.1002/anie.9624043

**Published:** 2026-06-04

**Authors:** Zhe Zhang, Xuanyu Nan, Zhengyu Huang, Jin Jin, Cheng Tian, Lian Chen, Xiaoli Hu, Huawei He, Yuhe Renee Yang, Cheng Zhi Huang, Chengde Mao, Hua Zuo

**Affiliations:** ^1^ Key Laboratory of Luminescence Analysis and Molecular Sensing (Southwest University) Ministry of Education College of Pharmaceutical Sciences Southwest University Chongqing China; ^2^ Yibin Academy of Southwest University Yibin China; ^3^ Laboratory of Nanosystem and Hierarchical Fabrication National Center for Nanoscience and Technology Beijing China; ^4^ State Key Laboratory of Chemical Resource Engineering Beijing University of Chemical Technology Beijing China; ^5^ Integrative Science Center of Germplasm Creation in Western China (Chongqing) Science City Biological Science Research Center Southwest University Chongqing China; ^6^ University of Chinese Academy of Sciences Beijing China; ^7^ Department of Chemistry Purdue University West Lafayette USA

**Keywords:** biomolecular nanostructures, DNA nanotechnology, protein‐DNA hybrid nanostructures, self‐assembly

## Abstract

DNA and proteins have been extensively explored for self‐assembly of nanostructures. Each has its own advantages and limitations. By integrating them together, protein‐DNA frameworks (PDFs) could potentially combine their advantages while overcoming the limitations associated with each component; thus, providing a huge diversity in terms of structures, functionalities, assembly versatilities, and responsiveness. Though each has been demonstrated to self‐assemble into large structures, it remains a great challenge to engineer well‐structured protein‐DNA interfaces for PDF assembly. Herein, we report a robust and versatile approach to this problem. Specific and high‐affinity protein‐aptamer binding can nicely interface protein and DNA with structural control. A series of bivalent thrombin aptamers were designed to co‐assemble with thrombin into a range of PDFs including discrete triangles and 3D prisms, linear and circular oligomers, 1D chains/ladders, and 2D arrays. The versatility of such a strategy was further demonstrated by the self‐assembly of *Plasmodium falciparum* lactate dehydrogenase (*Pf*LDH)—containing PDFs. In this work, AlphaFold 3, a universal modeling program, dramatically facilitates structural modeling and helps the designs. We believe that such a rational PDF design will find wide applications as multimodal biomaterials integrating the diverse functionalities of proteins and the ease of assembly programmability of DNA.

## Graphical abstract

Rationally designed DNA multivalent aptamers direct specific protein binding to drive self‐assembly into protein‐DNA frameworks (PDFs). Structurally defined protein‐aptamer interfaces organize proteins into triangles, linear and ladderlike chains, and periodic two‐dimensional arrays, enabling precise control over orientation, connectivity, and overall architecture.

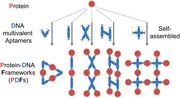

## 1 Introduction

Biomolecule self‐assembly is essential to many biological processes and plays a pivotal role in various technological applications including drug delivery, biosensing and imaging, nanodevices, and molecular computation. Both DNA and protein have been successfully programmed to assemble into a wide range of nanostructures [1, 2, 3, 4, 5, 6, 7, 8, 9]. DNA is well demonstrated for its superb capability for programmed self‐assembly [1, 2, 3, 4, 5, 6, 10, 11, 12, 13], but, in general, lacks technologically useful functions. On the other hand, proteins are rich in functions, but, challenging to be programmed to self‐assemble into desired structures in spite of recent impressive progress [7, 8, 9]. A natural question arises: can DNA and protein be engineered to co‐assemble in such a way that it integrates the advantages of both DNA and protein? Although this concept has been explored [14, 15, 16, 17, 18], very limited progress has been made in terms of both geometrically and orientationally well‐defined nanostructures [19, 20, 21, 22, 23, 24, 25, 26, 27, 28, 29, 30, 31, 32, 33, 34, 35, 36, 37, 38, 39, 40, 41, 42, 43, 44, 45]. One key challenge is how to interface DNA and proteins in a structurally predictable fashion [27, 28, 29, 30, 31, 32, 33, 34, 35, 36, 37, 38]. Interfacing protein‐DNA often involves flexible linker that is structurally uncontrolled. Such undesired flexible interfaces will likely lead to certain structural disorders (in terms of location and orientation) in the resulting hybrid nanostructures. Herein, we proposed a versatile approach for assembly of protein‐DNA frameworks (PDFs) by incorporating DNA aptamers that can specifically bind to proteins in a structurally well‐defined fashion [46]. The PDFs are co‐assembled from both DNA and proteins; no frameworks can form from any component alone. This strategy has been concretely demonstrated by successfully designing and assembling a range of geometrically well‐defined PDFs, including linear and circular oligomers, 1D chains, and 2D arrays, from two proteins and their corresponding aptamers, first thrombin (T), and then *Plasmodium falciparum* lactate dehydrogenase (*Pf*LDH). In this work, AlphaFold 3 [47], a universal modeling program, dramatically facilitates structural modeling and helps the designs. Compared with DNA origami, which typically relies on scaffold‐based folding and subsequent incorporation of proteins through various interactions (e.g., covalent conjugation [48, 49], noncovalent interactions [26, 50, 51, 52], and protein tagging approaches [53, 54]), DNA aptamers have also been widely integrated into DNA origami structures as functional nucleic acid elements for target recognition and protein detection [55, 56, 57, 58, 59, 60]. In these systems, aptamers generally serve as peripheral functional motifs that enable sensing or targeting, while the overall architecture is predefined by the DNA scaffold. In contrast, the present strategy directly integrates proteins into the structural framework via structurally defined aptamer‐protein interfaces, enabling proteins to function as intrinsic structural elements with controlled orientation and positioning while supporting efficient co‐assembly with near 1:1 DNA‐to‐protein stoichiometry. Rather than decorating preformed DNA architectures, our approach employs aptamer‐protein interactions as programmable and structurally defined connectors that guide the assembly process itself. While DNA origami excels at constructing highly complex architectures, our approach provides a complementary route that is particularly suited for building protein‐DNA assemblies with well‐defined interfaces and integrated structural and functional roles. We believe that this versatile approach would allow the development of PDFs, a new class of biomaterials, for a wide range of applications enabled by the structures and functions associated with both DNA and proteins.

## 2 Results and Discussion

**FIGURE 1:**
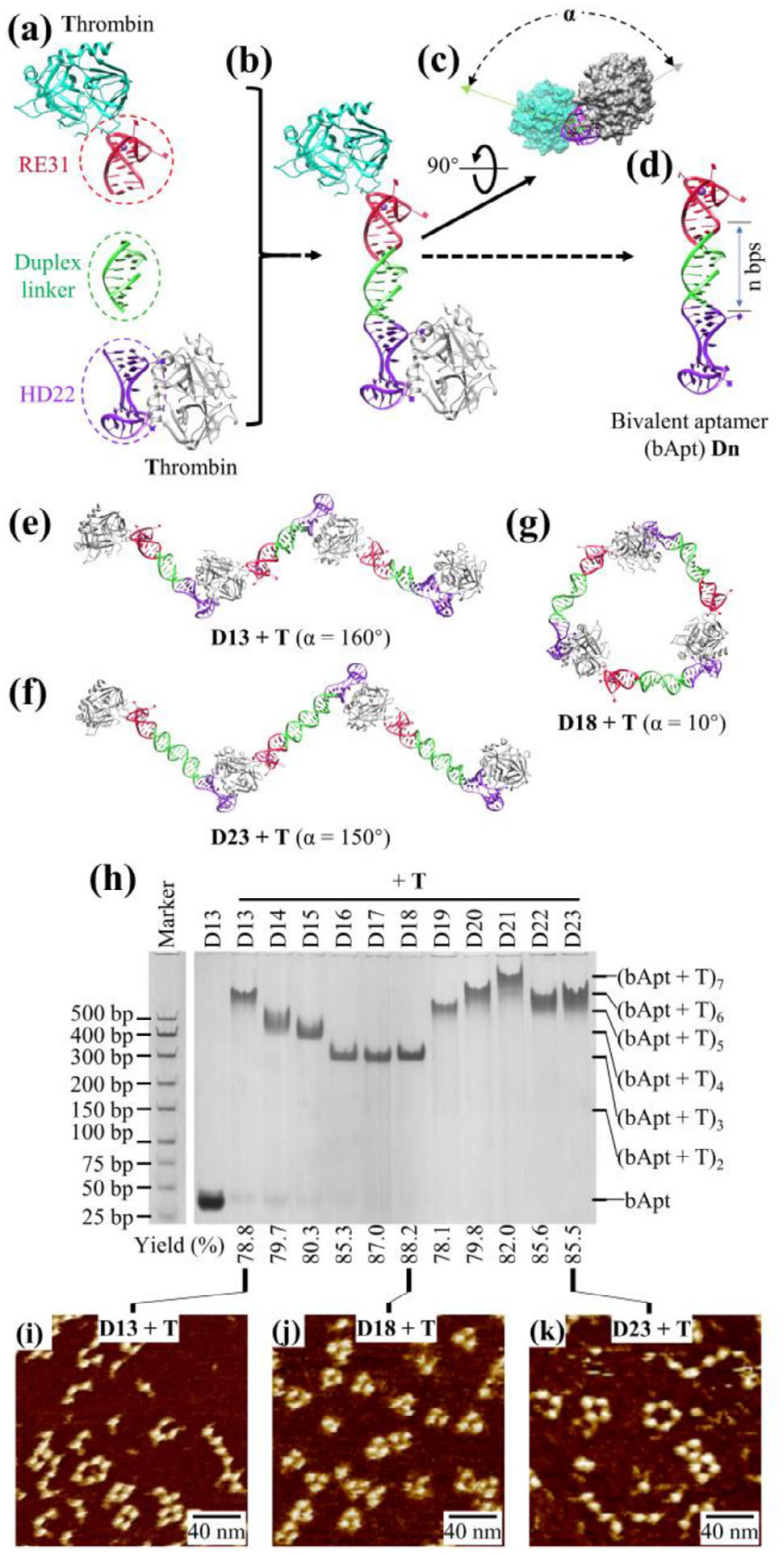
Self‐assembly of protein‐DNA frameworks (PDFs) from thrombin (T) protein and its bivalent DNA aptamer (bApt) Dn. (a–d) AlphaFold 3 (AF3)—aided design of Dn based on the crystal structures of two different aptamer‐T complexes (RE31‐T and HD22‐T) and a DNA duplex linker. *n*: the number of base pairs (pbs) between the two aptamers. *α*: dihedral angles between two thrombin molecules in T‐Dn‐T complexes. Note that *α* changes as n changes because of the helical nature of DNA duplexes. (e–g) Models of PDF oligomers as a function of *n*. (h) Native polyacrylamide gel electrophoresis (nPAGE, 4%) analysis of PDF oligomers. The yields shown below the gel correspond to the oligomers in each lane. (i–k) AFM images of three examples of assembled PDF oligomers.

We started with a simple question: can thrombin be programmed to assemble into oligomers by DNA? Two orthogonal DNA aptamers (HD22 and RE31) were previously reported to specifically bind to thrombin (T), a protein involved in thrombogenesis. The crystal structures of the thrombin‐aptamer complexes were available (PDB ID: 4i7y and 5cmx for HD22‐T complex and RE31‐T complex, respectively) [61, 62], and the two aptamers could simultaneously bind to different sites of thrombin (Figures 1a–c and S1a–1b) [63]. Based on the crystal structures, we designed bivalent aptamers (bApt) by joining RE31 and HD22 with rigid DNA duplex linkers. A bApt was named as Dn; *n* indicated the length (in base pairs, bps) of the DNA duplex linker (Figures 1d and S1c–1d). Upon equal molar mixing, Dn and T would associate with each other to form hetero‐oligomers with Dn and T alternatingly arranged. The overall structure of the oligomers would vary according to the bApt structures. To find out the relative orientation between two T molecules bound to the same bApt in a Dn‐T complex, we used AF3 to model the (Dn)_1_(T)_2_ complex (Figures S2a–2b). The modeled structures were further refined by using the crystal structures (PDB ID: 4i7y and 5cmx) to replace the corresponding portions in the model, resulting in final refined models of (Dn)_1_(T)_2_ (Figures S2c–2d).

From the (Dn)_1_(T)_2_ structure and the fused RE31‐T‐HD22 crystal structure, we could readily predict the oligomeric structures co‐assembled from Dn and T (Figures 1e–g and S3). In (D13)_1_(T)_2_, the dihedral angle (*α*) between T‐D13‐T is 160°, ∼ 180° (Figures 1e and S3a). The complex is nearly planar and the two thrombin molecules face opposite sides of D13. By equal molar mixing D13 and T, extended, zigzag chains of various lengths are expected, which could further cyclize into rings due to flexibility associated with the DNA and DNA‐protein interfaces. A similar situation will happen to the D23‐T assembly as D23 is extended from D13 by one helical turn, thus, has similar geometry (Figures 1f and S3c). However, the structure will change for D18‐T assembly (Figures 1g and S3b). D18 is extended from D13 by five bps, a half helical turn. Thus, the dihedral angle (*α*) between T‐D18‐T in a (D18)_1_(T)_2_ complex becomes 10°, ∼ 0°, allowing the two thrombin proteins to face to the same side of D18. As oligomerizing, any curvature will accumulate, promote D18‐T assembly to cyclize into cyclotrimer.

We experimentally tested these designs by native polyacrylamide gel electrophoresis (nPAGE, Figure 1h), showing that the Dn‐T complexes are formed with yields of 70%–90%. The size of the assembled complex is a function of the value of *n*, the length of the duplex linker between the two aptamer moieties in a bApt. The size varied in a wave form as the DNA duplex twisted with a periodicity of ∼ 10 bps (a full helical turn). Large complexes formed for D13‐T and D23‐T assembly (slow mobilities) and small complexes formed for D18‐T assembly (high mobility). The complex structures were further characterized by atomic force microscopy, AFM (Figures 1i–k and S4). Both D13‐T and D23‐T complexes appeared as linear chains and circles of different sizes. Due to the AFM sample preparation, the structures were partially disrupted. D18‐T complex was well‐defined triangles. Because of the small size and circle geometry, the triangles were resistant to the disruption during AFM sample preparation. We further characterized the self‐assembled structure of D17‐T complex using negative‐stain electron microscopy (ns‐EM), confirming its triangular geometry (Figure S4b). This series of assembled linear Dn‐T structures were flexible (prone to cyclize) and fragile. Dissociation of any Apt‐T binding would fragment Dn‐T chains, leading to short chain length.

**FIGURE 2:**
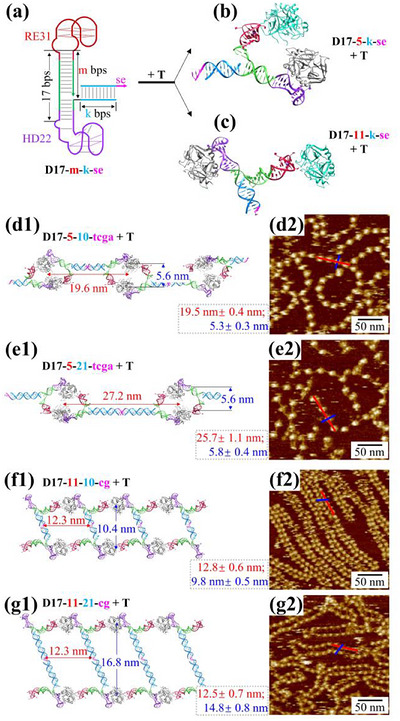
Robust, double‐connected 1D PDF arrays assembled from bApts with a side duplex branch and thrombin. (a) Scheme of the bApt with definition of each parameter. se: palindromic sticky end. (b–c) Structural models of the (bApt)_1_(T)_2_ complexes with different m values. Note the dramatic conformational change of the complexes. Structural models and corresponding AFM images for co‐assembly of T and D17‐5‐10‐tcga (d1, d2), D17‐5‐21‐tcga (e1, e2), D17‐11‐10‐cg (f1, f2) and D17‐11‐21‐cg (g1, g2). The measured values of the repeating distances/widths along the red/blue lines on AFM images are indicated on the left to the images. The expected values are calculated from and indicated on the models.

To increase the robustness and the range of accessible structures of the PDFs, we introduced a side duplex branch to the duplex linker region of D17 (Figures 2a and S1e). The side branch bears a self‐complementary sticky end to introduce additional interactions between the bApts; thus, increases PDF connectivity, which, in turn, enhances the PDF robustness. The new bApt contains three helical domains and is dubbed as D17‐m‐k‐se. Here, *m* denotes the length of the top helical domain, *k* represents the length of the side duplex branch, and se refers to the palindromic sequence of the sticky end on the side branch. The introduction of the side branch would cause the linker duplex to bend away from the side branch as a Y‐shaped geometry. Depending on the value of *m*, the bending would position the two bApt‐bound thrombin molecules to face to different directions and would allow assembly of two classes of robust 1D arrays at nanomolar concentrations. (Figures 2 and S5–S8).

When *m* = 5 (Figure 2b), the two thrombin molecules face to each other, leading to formation of a (bApt)_2_(T)_2_ complex, which can further associate with each other via hybridization between the sticky ends on the side branches (Figures 2d1 and 2e1). Eventually, 1D chains with different periodicities are formed and the corresponding beads‐on‐a‐string morphologies are clearly confirmed by AFM imaging (Figures 2d2 and 2e2). Each bead consists of two thrombin molecules. The inter‐beads distances can be readily varied by changing the length (*k*) of the side duplex branch. Two beads‐on‐a‐string chains have been assembled and the measured repeating distances closely matche the values calculated from the designs. When *m* = 11 (half turn away from *m* = 5) as shown in Figure 2c, bApt‐T first form singly connected chains, similar to the chains assembled from D18‐T. The side duplexes are located on the same side of the chain. Hybridization of sticky ends on the side duplexes will associate two singly connected chains together to form robust, doubly connected, ladderlike structures (Figures 2f1 and 2g1), which are confirmed by AFM imaging (Figures 2f2 and 2g2). Note that parallel, double connections along the 1D structures strengthen these two types of 1D structures (Figure 2), which grow much longer and stiffer than the singly connected chains assembled from D13‐T or D23‐T (Figure 1). To verify the specificity of PDF assembly, three control systems are designed based on the bApt D17‐5‐10‐tcga: (i) a scrambled sequence (RD‐10‐tcga) incubated with thrombin; (ii) a mutant aptamer (MD‐5‐10‐tcga) with mutations in the G‐quadruplex region critical for binding; and (iii) the bApt D17‐5‐10‐tcga incubated with a non‐target protein (human coagulation factor IXa (FIXa)). Under identical conditions, no defined assemblies are observed in any of these control systems by AFM imaging (Figure S9), confirming that the formation of PDFs is highly dependent on specific aptamer‐protein interactions.

**FIGURE 3:**
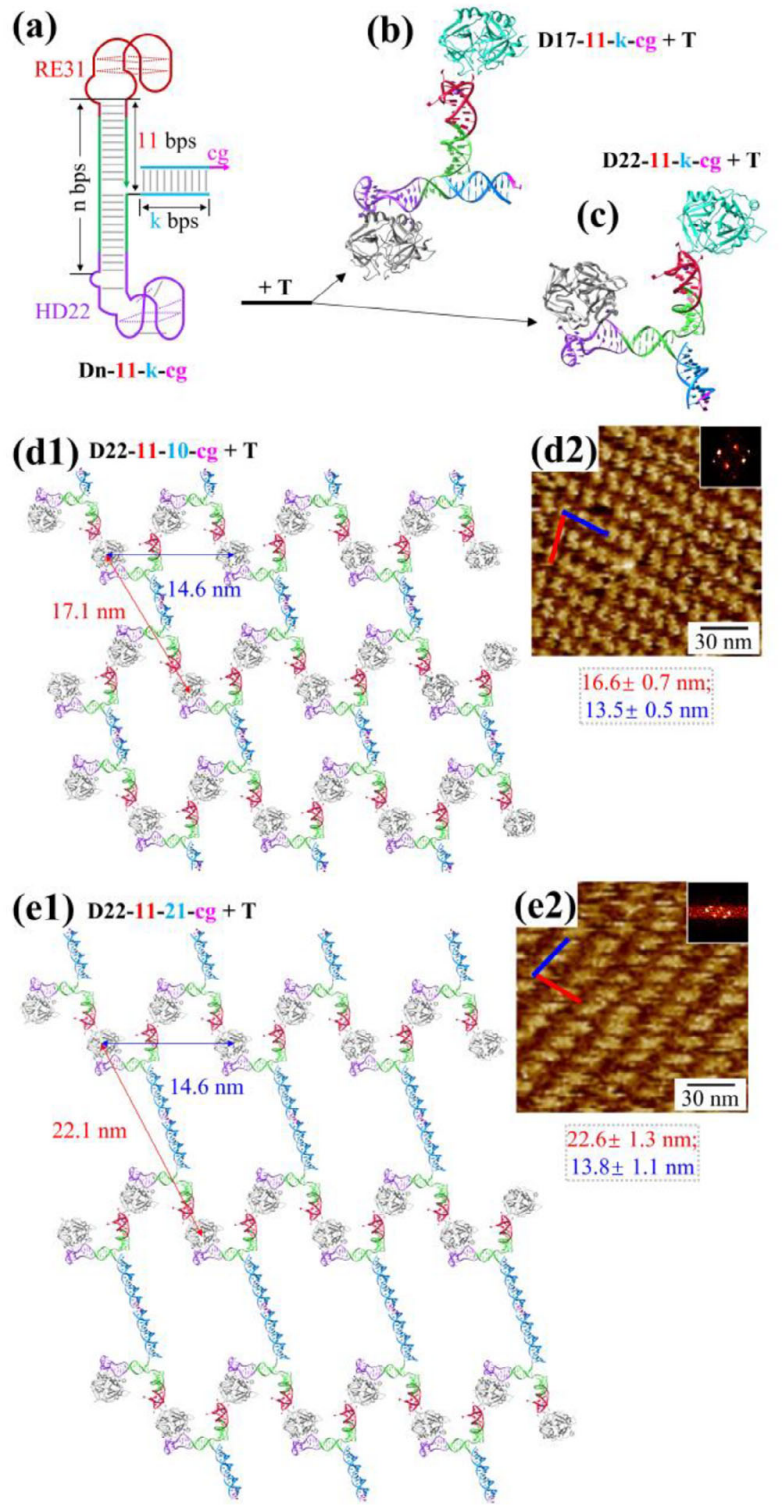
Assembly of PDF 2D arrays from bApt and thrombin. (a) The secondary structure of the component bApt Dn‐11‐k‐cg. (b–c) Structural models of the (bApt)_1_(T)_2_ complexes with different *n* values. Structural models (d1, e1) and corresponding AFM images (d2, e2) of the two PDF 2D arrays assembled from D22‐11‐k‐cg and thrombin. The two bApts have different *k* values, corresponding to different periodicities. The measured values of the repeating distances along the red/blue lines on AFM images are indicated below the images. The expected values are calculated from and indicated on the models.

This strategy can be further extended to design and assemble PDF 2D arrays (Figures 3 and S10, S11). In the above studies, we have identified two key parameters for assembly of bApt‐T PDF nanostructures: (i) dihedral angle (*α*) between T‐bApt‐T and (ii) the position of the side duplex branch in bApt. We have designed bApt D22‐11‐k‐cg by extending the lower helical domain of bApt D17‐11‐k‐cg by 5 bps (approximately a half helical turn), which adds a 180° rotation between the two aptamer binding faces (Figures 3b and 3c). When D22‐11‐k‐cg and thrombin assemble into 1D chains, the side duplex branches will be alternatingly located on the opposite sides of the chains (Figure S10). Via hybridization of sticky ends of the side duplex branches, each 1D chain can associate with another two 1D chains, one above and one below, leading to formation of periodic, PDF 2D arrays at nanomolar concentrations (Figures 3d1 and 3e1), which have been confirmed by AFM imaging (Figures 3d2, 3e2, and S10, S11). The observed repeating distances are consistent with the values calculated from the designs. By varying the side branch length, the repeating distance can be varied. Such periodicity changes indeed exist in AFM images.

**FIGURE 4:**
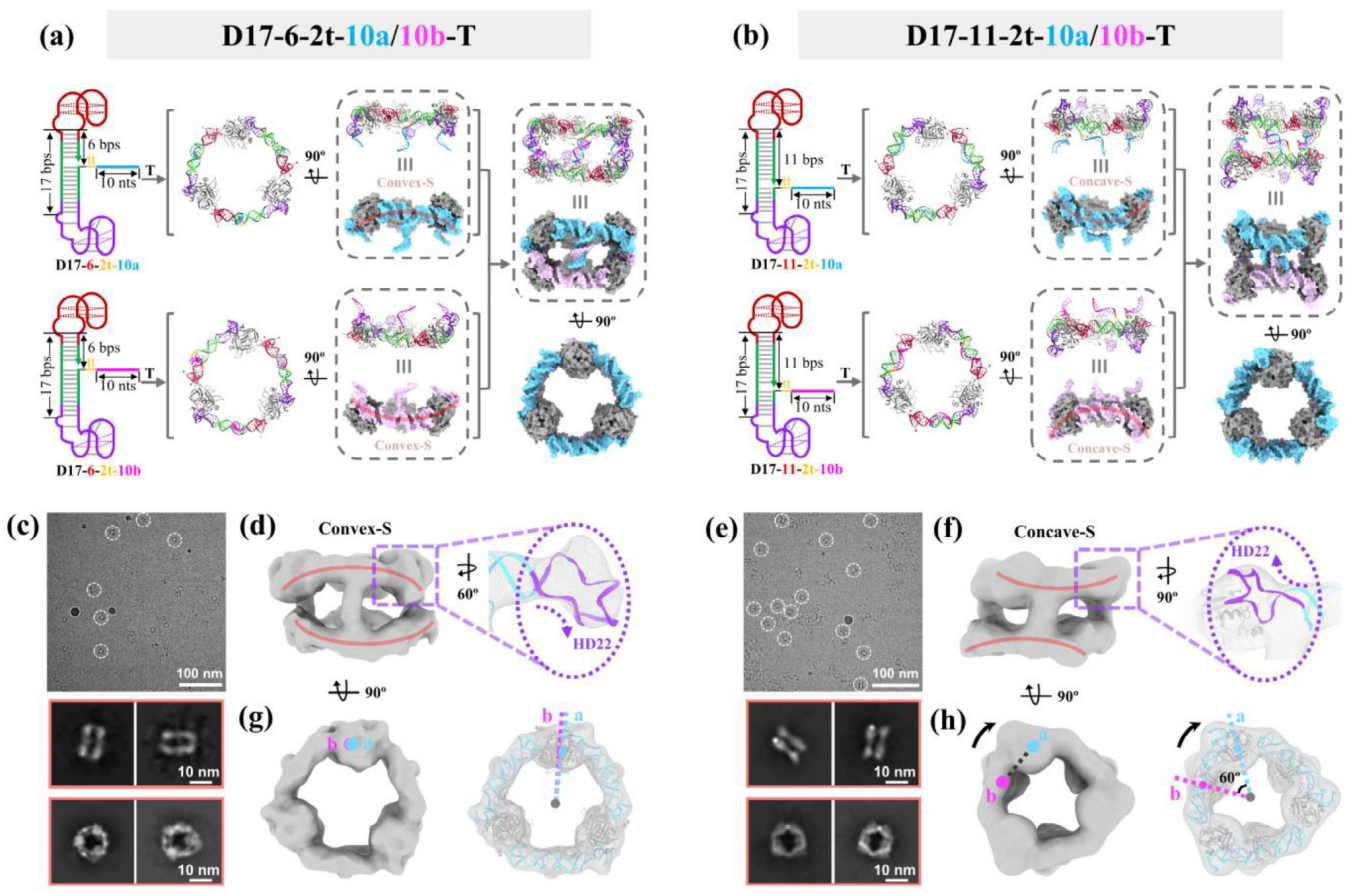
Assembly of PDF nanocages from bApts and thrombin. (a, b) Scheme of the assembly process. Two bApts, D17‐6‐2t‐10a and D17‐6‐2t‐10b (a), or D17‐11‐2t‐10a and D17‐11‐2t‐10b (b), each assemble with thrombin into triangles with complementary tails. Upon hybridization between the tails, two triangles associate into a trigonal PDF prism. The two surfaces of the triangles are named based on their geometry as the convex surface (Convex‐S) and the concave surface (Concave‐S). (c) Cryo‐EM analysis of D17‐6‐2t‐10a/10b‐T. Representative raw micrographs and 2D class averages are displayed. (d) The 3D reconstruction of D17‐6‐2t‐10a/10b‐T based on Cryo‐EM is displayed from the side view. The pseudoatomic model is fitted into the density map, with the HD22 aptamer orientation angle highlighted. (e) Cryo‐EM analysis of D17‐11‐2t‐10a/10b‐T. Representative raw micrographs and 2D class averages are displayed. (f) The 3D reconstruction of D17‐11‐2t‐10a/10b‐T based on Cryo‐EM is displayed from the side view. The pseudoatomic model is fitted into the density map, with the HD22 aptamer orientation angle highlighted. (g, h) The 3D reconstructions of D17‐6‐2t‐10a/10b‐T (g) and D17‐11‐2t‐10a/10b‐T (h) based on Cryo‐EM are displayed from the top view. The pseudoatomic model is fitted into the density map, with the twist angle between the two triangular layers labeled.

Trigonal prisms, as PDF 3D nanocages, have been assembled by association of two triangles. We modified D17 by adding a single‐stranded tail to the duplex linker and dubbed the new bApts as D17‐m‐2t‐10a and ‐10b (Figures 4a and 4b). *m* indicates the length of the top helical domain; 2t stands for a TT spacer; 10a and 10b stand for a pair of 10‐base‐long, complementary sequences (Figures 4a and 4b). Each of these bApts can separately co‐assemble with thrombin into trimeric triangles, similar to D17‐T complex. Upon mixing the two different triangles, hybridization between the complementary single‐stranded tails will bring the two triangles together to form a hexameric trigonal PDF prism (Figures 4a and 4b). Both nPAGE and single‐particle cryo‐electron microscopy (cryo‐EM) imaging demonstrated the successful assembly of trigonal PDF prisms with yields of 70%–90% (Figures 4c–h and S12–S14). Cryo‐EM imaging revealed monodisperse particles with the expected dimensions and trigonal prism geometry (Figures 4c and 4e), allowing us to reconstruct the 3D structure without reference, with overall resolutions of 10.63 Å (D17‐6‐2t‐10a/10b‐T) and 18.61 Å (D17‐11‐2t‐10a/10b‐T) (Figure S13, S14, Table S1, S2). We first fitted the RE31‐T‐HD22 crystal structure as the starting unit into the cryo‐EM density map, followed by fitting of the linker and triangular duplex regions and connecting all components to obtain a pseudoatomic model covering the entire structure (Figure S15). Please note that the PDF triangle has two distinct surfaces. By selecting an appropriate m value (6 or 11 base pairs), the single‐stranded tails will be positioned on either the convex or concave surface of the triangle (Figures 4a–b). Dimerization of these triangles results in two distinct conformations of trigonal PDF prisms, which exhibit different electrophoretic mobilities as observed in nPAGE (Figures S12). Cryo‐EM analysis further confirms the structural differences between the two forms of trigonal PDF prisms. Specifically, at *m* = 6, the single‐stranded tails are oriented towards the HD22‐down surface, forming a trigonal PDF prism with convex triangular layers (Figures 4c, d). In contrast, at *m* = 11, the tails are positioned on the HD22‐up surface, resulting in concave triangular layers (Figures 4e, f). Additionally, Cryo‐EM analysis reveals that the two triangular layers of D17‐6‐2t‐10a/10b‐T are well‐aligned, consistent with the AF3‐aided prediction model (Figures 4a and 4g). However, owing to the length‐constrained linker, D17‐11‐2t‐10a/10b‐T exhibits an ∼60° interlayer angular offset that alleviates geometric constraints and adopts an entropically favored conformation, thereby deviating from the AF3‐aided prediction model (Figures 4b and 4h).

**FIGURE 5:**
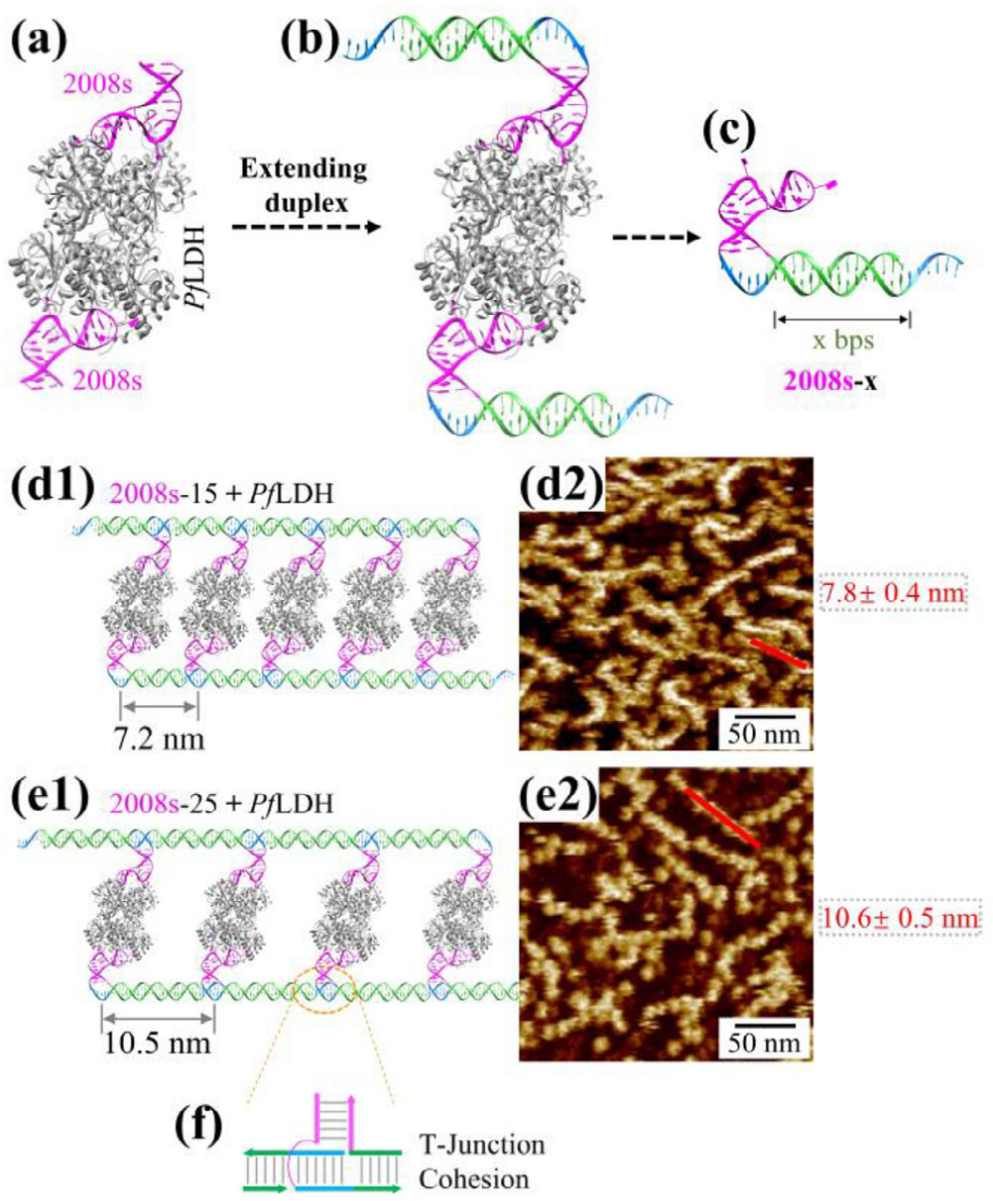
PDF chains co‐assembled by *Plasmodium falciparum* lactate dehydrogenase (*Pf*LDH) and its aptamer 2008s‐x. (a) Crystal structure of the complex of homo‐tetrameric *Pf*LDH and DNA aptamer 2008s. (b) Extra DNA components are added to aptamer 2008s in the crystal structure. (c) Modified aptamer 2008s‐x contains an x‐bp‐long duplex, a pair of complementary 6‐base‐long bulge loop and a tail. (b–c) Refined models of 2008s‐x + *Pf*LDH and 2008s‐x. (d–e) PDF chains co‐assembled from 2008s‐x and *Pf*LDH. (d1), (e1) Structural models; (d2), (e2) AFM images. (f) Scheme of T‐junction cohesion between 2008s‐x via hybridization between the bulge loop and the tail. The measured values of the repeating distances along the chains on AFM images are indicated on the right of the AFM images and the expected values are calculated from and indicated on the models.

The aptamer‐based strategy for PDF assembly is a versatile approach as long as a protein or protein complex can simultaneously bind to two DNA aptamers. There are at least two applicable situations: (i) a protein (or protein complex), such as thrombin, can simultaneously bind to two different aptamers, and (ii) a protein homomer can bind to two identical aptamers. Given that most proteins form symmetric homomers [64], the latter would provide many opportunities for this strategy. As a demonstration, we examined *Plasmodium falciparum* lactate dehydrogenase (*Pf*LDH) and its DNA aptamer 2008s (Figures 5 and S16–S19) [65]. *Pf*LDH forms a homo‐tetramer with a tetrahedral symmetry. A crystallography study shows that the *Pf*LDH tetramer binds to two 2008s aptamers as shown in Figure 5a (PDB ID: 3ZH2). Based on *Pf*LDH‐2008s complex, we computationally extended both 5’ and 3’ ends to the aptamers (Figures 5b and 5c). The extended regions are parallel to each other and consist of a x‐bp‐long helical domain, a 6‐base‐long tail, and a 6‐base‐long bulge loop. The sequences of the tail and the bulge loop are complementary to each other. The modified aptamers are named 2008s‐x; *x* indicates the length of the added helical domain. Aptamers 2008s‐x can associate with one another via T‐junction cohesion (Figure 5f), which is formed by hybridization between the tail and the bulge loop. Eventually, PDF ladders will be assembled with continuous DNA duplexes as side rails and *Pf*LDH‐2008s as rungs (Figures 5d1 and 5e1). The separation between the rungs can be readily adjusted by varying the length of the extended helical domain. After assembly, the PDF samples were analyzed by AFM and nPAGE. As expected, the PDF ladders were clearly observed and the apparent repeating distances were well consistent with the values calculated from the designs, with yields of approximately 70% (Figures 5d2, 5e2, and S18, S19).

## 3 Conclusion

In conclusion, we have developed a rational, versatile approach for self‐assembly PDF nanostructures based on protein‐aptamer binding. The protein‐DNA interface is structurally well defined, which is in sharp contrast to many other studies, where protein‐DNA interfaces often involve flexible linkers. The approach has been experimentally demonstrated with two orthogonal protein systems. In general, the extension of this strategy to other systems relies on several key requirements. First, the aptamer‐protein interaction should exhibit sufficiently high affinity, ideally with *K*_d_ value in the nanomolar range, to ensure stable and efficient binding under equilibrium conditions, which is essential for driving ordered assembly. Second, the target protein should possess two or more independent aptamer‐binding sites, enabling it to function as a connecting node for network formation and thus support the construction of higher‐order architectures. Third, the non‐binding regions of the aptamer should be amenable to sequence and structural extension, allowing the incorporation of additional DNA motifs or domains that can interface with other components in the framework. However, these requirements can be readily achieved using the well‐established SELEX (systematic evolution of ligands by exponential enrichment) technology for protein‐targeted aptamer selection [66, 67]. With this development, it becomes possible to incorporate the vast available structures and functionalities of both DNA and protein, two prominent classes of biomolecules, into one integrated molecular system. Both DNA and proteins are integral components, instead of pendants, of the PDFs; thus, both molecules are well‐defined in both location and orientation. Such precise structural control is essential for engineering high performance molecular machines to imitate natural nucleoproteins (e.g. ribosomes) in cells. Beyond structural design, these protein‐DNA frameworks provide opportunities for functional applications that require precise spatial organization of proteins. In particular, they may serve as platforms for ordered enzyme immobilization and multienzyme cascade reactions, as exemplified by the *Pf*LDH‐based system, as well as for multivalent protein display and target capture enabled by programmable spatial arrangement. In addition, the ability to position proteins with defined geometry may be further exploited in applications such as multivalent biosensing and programmable antigen display. The PDFs therefore represent a new class of materials that integrate the programmability of DNA with the functionality of proteins, providing a foundation for the development of advanced biomolecular systems.

## Author Contributions

**Zhe Zhang** and **Xuanyu Nan** contributed equally to this work. Zhe Zhang performed the structural design, AFM scanning, PAGE characterization, data analysis, and drafted the manuscript. Xuanyu Nan and **Yuhe Renee Yang** performed and analyzed the ns‐EM and cryo‐EM structures. **Zhengyu Huang** and **Huawei He** expressed and purified *Pf*LDH and investigated its interaction with the DNA aptamer. **Jin Jin** and **Cheng Tian** conducted part of AFM scanning and nPAGE characterization. **Lian Chen** assisted Zhengyu Huang with PfLDH expression and purification. **Xiaoli Hu** performed AFM scanning of the PDF oligomers. **Hua Zuo**, **Cheng Zhi Huang**, and **Chengde Mao** led and supervised the project, initiated the concept and design, and revised the manuscript. All authors comment on the manuscript.

## Funding

This work was supported by National Natural Science Foundation of China (22274135 to H. Zuo and 22134005 to C. Z. Huang), Natural Science Foundation of Chongqing, China (CSTB2024NSCQ‐ MSX0907 to H. Zuo, CSTB2022NSCQ‐LZX0302 to H. W. He, and CSTB2024TIAD‐CYKJCXX0039 to C. Z. Huang), National Key Research and Development Program of China (2022YFD1201600 to H. W. He), the National Science Foundation (CCF2107393 to C. D. Mao), National Key R&D Program of China (2022YFA1206400 to Y. R. Yang), Beijing Natural Science Foundation (F251001 to Y. R. Yang), National Natural Science Foundation of China (22277017 to Y. R. Yang) and Strategic Priority Research Program of Chinese Academy of Sciences (XDB0770000 to Y. R. Yang).

## Conflicts of Interest

The authors declare no conflicts of interest.

## Supporting information




**Supporting Information**: anie72825‐sup‐0001‐SuppMat.docx.
Figure S1: List of the secondary structures of all thrombin bApts. Figure S2: AF3‐aided modeling process. Figure S3: AF3‐aided prediction of oligomeric structures. Figure S4: Images of oligomeric structures. Figure S5: Double‐connected PDF 1D arrays assembled from D17‐m‐10‐tcga (*m* = 6, 7) and T. Figure S6: Double‐connected PDF 1D ladders assembled from D17‐m‐10‐cg (*m* = 8–10) and T. Figure S7: Double‐connected PDF 1D arrays assembled from D17‐m‐k‐se (*m* = 5 or 11; *k* = 10 or 21) and T. Figure S8: AFM images for co‐assembly of T and D17‐5‐10‐tcga (a) and D17‐11‐10‐cg (b) at different concentrations. Figure S9: AFM images of assemblies formed by T with random sequence (RD‐10‐tcga) and mutant sequence (MD‐5‐10‐tcga), and the assembly of D17‐5‐10‐tcga with FIXa. Figure S10: 2D arrays assembled from D22‐11‐k‐cg (*k* = 10 or 21) and T. Figure S11: AFM images for co‐assembly of T and D22‐11‐10‐cg at different concentrations. Figure S12: nPAGE (4%) characterization of the trigonal PDF prisms assembled from D17‐m‐2t‐10a/10b (*m* = 6–11) and T. Figure S13: Cryo‐EM data processing workflow for the D17‐6‐2t‐10a/10b‐T. Figure S14: Cryo‐EM data processing workflow for the D17‐11‐2t‐10a/10b‐T. Figure S15: Workflow for constructing the RE31‐T‐HD22‐based trigonal PDF prisms models fitted into the cryo‐EM density maps. Figure S16: Map of the recombinant plasmid encoding pET28a‐*Pf*LDH. Figure S17: SDS‐PAGE analysis of the *Pf*LDH protein. Figure S18: nPAGE (4%) characterization of the PDF chains co‐assembled by *Pf*LDH and 2008s‐x (x = 15 or 25). Figure S19: PDF chains co‐assembled by *Pf*LDH and its aptamer 2008s‐x. Table S1: Cryo‐EM Data collection parameters of D17‐6‐2t‐10a/10b‐T. Table S2: Cryo‐EM Data collection parameters of D17‐11‐2t‐10a/10b‐T.

## Data Availability

The data that supports the findings of this study are available in the supplementary material of this article.

## References

[1] N. C. Seeman and H. F. Sleiman , “DNA Nanotechnology,” Nature Reviews Materials 3 (2018): 17068, 10.1038/natrevmats.2017.68.

[2] M. R. Jones , N. C. Seeman , and C. A. Mirkin , “Programmable Materials and the Nature of the DNA Bond,” Science 347 (2015): 1260901, 10.1126/science.1260901. 25700524

[3] F. Hong , F. Zhang , Y. Liu , and H. Yan , “DNA Origami: Scaffolds for Creating Higher Order Structures,” Chemical Reviews 117 (2017): 12584–12640, 10.1021/acs.chemrev.6b00825. 28605177

[4] A. V. Pinheiro , D. R. Han , W. M. Shih , and H. Yan , “Challenges and Opportunities for Structural DNA Nanotechnology,” Nature Nanotechnology 6 (2011): 763–772, 10.1038/nnano.2011.187. PMC3334823 22056726

[5] H. Ramezani and H. Dietz , “Building Machines With DNA Molecules,” Nature Reviews Genetics 21 (2020): 5–26, 10.1038/s41576-019-0175-6. PMC6976304 31636414

[6] Y. H. Dong , C. Yao , Y. Zhu , L. Yang , D. Luo , and D. Y. Yang , “DNA Functional Materials Assembled From Branched DNA: Design, Synthesis, and Applications,” Chemical Reviews 120 (2020): 9420–9481, 10.1021/acs.chemrev.0c00294. 32672036

[7] J. Zhu , N. Avakyan , A. Kakkis , et al., “Protein Assembly by Design,” Chemical Reviews 121 (2021): 13701–13796. 34405992 10.1021/acs.chemrev.1c00308 PMC9148388

[8] S. L. Kuan , F. R. G. Bergamini , and T. Weil , “Functional Protein Nanostructures: A Chemical Toolbox,” Chemical Society Reviews 47 (2018): 9069–9105, 10.1039/C8CS00590G. 30452046 PMC6289173

[9] Z. Li , S. Z. Wang , U. Nattermann , et al., “Accurate Computational Design of Three‐Dimensional Protein Crystals,” Nature Materials 22 (2023): 1556–1563, 10.1038/s41563-023-01683-1. 37845322 PMC12253981

[10] T. Q. Zhang , X. M. Qian , W. W. Zeng , and B. Y. Wei , “Custom Folding of Double‐Stranded DNA Directed by Triplex Formation,” Chem 9 (2023): 1505–1517.

[11] C. Ng , A. Samanta , O. A. Mandrup , et al., “Folding Double‐Stranded DNA Into Designed Shapes With Triplex‐Forming Oligonucleotides,” Advanced Materials 35 (2023): 2302497, 10.1002/adma.202302497. 37311656

[12] G. Posnjak , X. Yin , P. Butler , et al., “Diamond‐Lattice Photonic Crystals Assembled From DNA Origami,” Science 384 (2024): 781–785, 10.1126/science.adl2733. 38753795 PMC7616107

[13] H. Liu , M. Matthies , J. Russo , et al., “Inverse Design of a Pyrochlore Lattice of DNA Origami Through Model‐Driven Experiments,” Science 384 (2024): 776–781, 10.1126/science.adl5549. 38753798

[14] N. Stephanopoulos , “Hybrid Nanostructures From the Self‐Assembly of Proteins and DNA,” Chem 6 (2020): 364–405.

[15] A. Hernandez‐Garcia , “Strategies to Build Hybrid Protein–DNA Nanostructures,” Nanomaterials 11 (2021): 1332, 10.3390/nano11051332. 34070149 PMC8158336

[16] C. Teller and I. Willner , “Organizing Protein–DNA Hybrids as Nanostructures With Programmed Functionalities,” Trends in Biotechnology 28 (2010): 619–628, 10.1016/j.tibtech.2010.09.005. 21035218

[17] J. B. M. Cluskey , D. S. Clark , and D. J. Glover , “Functional Applications of Nucleic Acid–Protein Hybrid Nanostructures,” Trends in Biotechnology 38 (2020): 976–989, 10.1016/j.tibtech.2020.02.007. 32818445

[18] K. Zhou , J. Y. Dong , Y. H. Zhou , J. C. Dong , M. Wang , and Q. B. Wang , “Toward Precise Manipulation of DNA–Protein Hybrid Nanoarchitectures,” Small 15 (2019): 1804044, 10.1002/smll.201804044. 30645016

[19] J. Procyk , E. Poppleton , and P. Sulc , “Coarse‐Grained Nucleic Acid–Protein Model for Hybrid Nanotechnology,” Soft Matter 17 (2021): 3586–3593, 10.1039/D0SM01639J. 33398312

[20] H. Al‐Zarah , M. F. Serag , M. Abadi , and S. Habuchi , “Self‐Assembly of Geometry‐Based DNA Origami‐Histone Protein Hybrid Nanostructures for Constructing Rationally‐Designed Higher‐Order Structures,” Acs Applied Nano Materials 6 (2023): 9515–9522, 10.1021/acsanm.3c01185.

[21] H. Al‐Zarah , M. F. Serag , F. Alkhaldi , and S. Habuchi , “A Versatile Approach for Geometry‐Based Self‐Assembly of DNA–Protein Hybrid Nanostructures Using Histone–DNA Interactions,” Chemical Communications 61 (2025): 532–535, 10.1039/D4CC05253F. 39648987

[22] Y. Xu , S. X. Jiang , C. R. Simmons , et al., “Tunable Nanoscale Cages From Self‐Assembling DNA and Protein Building Blocks,” ACS Nano 13 (2019): 3545–3554, 10.1021/acsnano.8b09798. 30835439

[23] J. D. Brodin , E. Auyeung , and C. A. Mirkin , “DNA‐Mediated Engineering of Multicomponent Enzyme Crystals,” Proceedings of the National Academy of Sciences of the United States of America 112 (2015): 4564–4569, 10.1073/pnas.1503533112. 25831510 PMC4403210

[24] P. H. Winegar , C. A. Figg , M. H. Teplensky , N. Ramani , and C. A. Mirkin , “Modular Nucleic Acid Scaffolds for Synthesizing Monodisperse and Sequence‐Encoded Antibody Oligomers,” Chem 8 (2022): 3018–3030. 36405374 10.1016/j.chempr.2022.07.003 PMC9674055

[25] O. G. Hayes , B. E. Partridge , and C. A. Mirkin , “Encoding Hierarchical Assembly Pathways of Proteins With DNA,” Proceedings of the National Academy of Sciences of the United States of America 118 (2021): e2106808118, 10.1073/pnas.2106808118. 34593642 PMC8501830

[26] A. A. Rafat , S. Sagredo , M. Thalhammer , and F. C. Simmel , “Barcoded DNA Origami Structures for Multiplexed Optimization and Enrichment of DNA‐Based Protein‐Binding Cavities,” Nature Chemistry 12 (2020): 852–859, 10.1038/s41557-020-0504-6. PMC7116572 32661410

[27] C. M. Niemeyer , T. Sano , C. L. Smith , and C. R. Cantor , “Oligonucleotide‐Directed Self‐Assembly of Proteins: Semisynthetic DNA—Streptavidin Hybrid Molecules as Connectors for the Generation of Macroscopic Arrays and the Construction of Supramolecular Bioconjugates,” Nucleic Acids Research 22 (1994): 5530–5539, 10.1093/nar/22.25.5530. 7530841 PMC310113

[28] D. Kashiwagi , S. Sim , T. Niwa , H. Taguchi , and T. Aida , “Protein Nanotube Selectively Cleavable With DNA: Supramolecular Polymerization of “DNA‐Appended Molecular Chaperones”,” Journal of the American Chemical Society 140 (2018): 26–29, 10.1021/jacs.7b09892. 29226681

[29] D. Kashiwagi , H. K. Shen , S. Sim , et al., “Molecularly Engineered “Janus GroEL”: Application to Supramolecular Copolymerization With a Higher Level of Sequence Control,” Journal of the American Chemical Society 142 (2020): 13310–13315, 10.1021/jacs.0c05937. 32691585

[30] H. K. Shen , K. Morishita , P. K. Hashim , et al., “ATP‐Responsive Nanoparticles Covered With Biomolecular Machine “Chaperonin GroEL”,” Angewandte Chemie International Edition 62 (2023): e202304894, 10.1002/anie.202304894. 37243902

[31] T. Teng , J. Bernal‐Chanchavac , N. Stephanopoulos , and C. E. Castro , “Construction of Reconfigurable and Polymorphic DNA Origami Assemblies With Coiled‐Coil Patches and Patterns,” Advanced Science 11 (2024): 2307257, 10.1002/advs.202307257. 38459678 PMC11132032

[32] A. Buchberger , C. R. Simmons , N. E. Fahmi , R. Freeman , and N. Stephanopoulos , “Hierarchical Assembly of Nucleic Acid/Coiled‐Coil Peptide Nanostructures,” Journal of the American Chemical Society 142 (2020): 1406–1416, 10.1021/jacs.9b11158. 31820959

[33] F. Praetorius and H. Dietz , “Self‐Assembly of Genetically Encoded DNA‐Protein Hybrid Nanoscale Shapes,” Science 355 (2017): eaam5488, 10.1126/science.aam5488. 28336611

[34] K. Zhou , Y. H. Zhou , V. Pan , Q. B. Wang , and Y. G. Ke , “Programming Dynamic Assembly of Viral Proteins with DNA Origami,” Journal of the American Chemical Society 142 (2020): 5929–5932, 10.1021/jacs.9b13773. 32191463

[35] C. Zhang , C. Tian , F. Guo , Z. Liu , W. Jiang , and C. D. Mao , “DNA‐Directed Three‐Dimensional Protein Organization,” Angewandte Chemie International Edition 51 (2012): 3382–3385, 10.1002/anie.201108710. 22374892

[36] Y. He , Y. Tian , A. E. Ribbe , and C. D. Mao , “Antibody Nanoarrays With a Pitch of ∼20 Nanometers,” Journal of the American Chemical Society 128 (2006): 12664–12665, 10.1021/ja065467+. 17002357

[37] Q. Y. Lu , Y. Xu , E. Poppleton , et al., “DNA‐Nanostructure‐Guided Assembly of Proteins Into Programmable Shapes,” Nano Letters 24 (2024): 1703–1709, 10.1021/acs.nanolett.3c04497. 38278134 PMC10853956

[38] M. X. You , R. W. Wang , X. B. Zhang , et al., “Photon‐Regulated DNA‐Enzymatic Nanostructures by Molecular Assembly,” ACS Nano 5 (2011): 10090–10095, 10.1021/nn204007y. 22098552 PMC3246559

[39] G. Grossi , M. D. E. Jepsen , J. Kjems , and E. S. Andersen , “Control of Enzyme Reactions by a Reconfigurable DNA Nanovault,” Nature Communications 8 (2017): 992, 10.1038/s41467-017-01072-8. PMC5648847 29051565

[40] S. P. Li , Q. Jiang , S. L. Liu , et al., “A DNA Nanorobot Functions as a Cancer Therapeutic in Response to a Molecular Trigger in Vivo,” Nature Biotechnology 36 (2018): 258–264, 10.1038/nbt.4071. 29431737

[41] P. Ketterer , A. N. Ananth , D. S. L. Trip , et al., “DNA Origami Scaffold for Studying Intrinsically Disordered Proteins of the Nuclear Pore Complex,” Nature Communications 9 (2018): 902, 10.1038/s41467-018-03313-w. PMC5834454 29500415

[42] J. L. Fu , Y. R. Yang , A. Johnson‐Buck , et al., “Multi‐Enzyme Complexes on DNA Scaffolds Capable of Substrate Channelling With an Artificial Swinging Arm,” Nature Nanotechnology 9 (2014): 531–536, 10.1038/nnano.2014.100. 24859813

[43] C. J. Delebecque , A. B. Lindner , P. A. Silver , and F. A. Aldaye , “Organization of Intracellular Reactions With Rationally Designed RNA Assemblies,” Science 333 (2011): 470–474, 10.1126/science.1206938. 21700839

[44] R. Freeman , M. Han , Z. Alvarez , et al., “Reversible Self‐Assembly of Superstructured Networks,” Science 362 (2018): 808–813, 10.1126/science.aat6141. 30287619 PMC6420308

[45] Q. Z. Feng , M. Saladin , C. X. Wu , et al., “Channel Width Modulates the Permeability of DNA Origami–Based Nuclear Pore Mimics,” Science Advances 10 (2024): eadq8773, 10.1126/sciadv.adq8773. 39536094 PMC11559598

[46] H. X. Yu , O. Alkhamis , J. Canoura , Y. Z. Liu , and Y. Xiao , “Advances and Challenges in Small‐Molecule DNA Aptamer Isolation, Characterization, and Sensor Development,” Angewandte Chemie International Edition 60 (2021): 16800–16823, 10.1002/anie.202008663. 33559947 PMC8292151

[47] J. Abramson , J. Adler , J. Dunger , et al., “Accurate Structure Prediction of Biomolecular Interactions With AlphaFold 3,” Nature 630 (2024): 493–500. 38718835 10.1038/s41586-024-07487-w PMC11168924

[48] J. Spratt , J. M. Dias , C. Kolonelou , et al., “Multivalent Insulin Receptor Activation Using Insulin–DNA Origami Nanostructures,” Nature Nanotechnology 19 (2024): 237–245, 10.1038/s41565-023-01507-y. PMC10873203 37813939

[49] L. Li , J. Yin , W. Ma , et al., “A DNA Origami Device Spatially Controls CD95 Signalling to Induce Immune Tolerance in Rheumatoid Arthritis,” Nature Materials 23 (2024): 993–1001, 10.1038/s41563-024-01865-5. 38594486

[50] J. L. Zhang , Y. Y. Xu , M. Y. Chen , et al., “Elucidating the Effect of Nanoscale Receptor‐Binding Domain Organization on SARS‐CoV‐2 Infection and Immunity Activation With DNA Origami,” Journal of the American Chemical Society 144 (2022): 21295–21303, 10.1021/jacs.2c09229. 36356984

[51] W. Engelen , C. Sigl , K. Kadletz , E. M. Willner , and H. Dietz , “Antigen‐Triggered Logic‐Gating of DNA Nanodevices,” Journal of the American Chemical Society 143 (2021): 21630–21636, 10.1021/jacs.1c09967. 34927433 PMC8719334

[52] S. C. M. Reinhardt , L. A. Masullo , I. Baudrexel , et al., “Ångström‐Resolution Fluorescence Microscopy,” Nature 617 (2023): 711–716, 10.1038/s41586-023-05925-9. 37225882 PMC10208979

[53] Q. Shen , T. Tian , Q. C. Xiong , et al., “DNA‐Origami NanoTrap for Studying the Selective Barriers Formed by Phenylalanine‐Glycine‐Rich Nucleoporins,” Journal of the American Chemical Society 143 (2021): 12294–12303, 10.1021/jacs.1c05550. 34324340 PMC8363578

[54] G. L. Ke , M. H. Liu , S. X. Jiang , et al., “Directional Regulation of Enzyme Pathways Through the Control of Substrate Channeling on a DNA Origami Scaffold,” Angewandte Chemie International Edition 55 (2016): 7483–7486, 10.1002/anie.201603183. 27159899

[55] X. X. Hu , H. L. Chi , X. Y. Fu , et al., “Tunable Multivalent Aptamer‐Based DNA Nanostructures to Regulate Multiheteroreceptor‐Mediated Tumor Recognition,” Journal of the American Chemical Society 146 (2024): 2514–2523, 10.1021/jacs.3c10704. 38247135

[56] S. Zhao , R. Tian , J. Wu , et al., “A DNA Origami‐Based Aptamer Nanoarray for Potent and Reversible Anticoagulation in Hemodialysis,” Nature Communications 12 (2021): 358, 10.1038/s41467-020-20638-7. PMC7807036 33441565

[57] W. T. Tang , T. Tong , H. Wang , et al., “A DNA Origami‐Based Gene Editing System for Efficient Gene Therapy in Vivo,” Angewandte Chemie International Edition 62 (2023): e202315093, 10.1002/anie.202315093. 37906116

[58] M. Raveendran , A. J. Lee , R. Sharma , C. Wälti , and P. Actis , “Rational Design of DNA Nanostructures for Single Molecule Biosensing,” Nature Communications 11 (2020): 4384, 10.1038/s41467-020-18132-1. PMC7463249 32873796

[59] M. Mao , Z. Lin , L. Chen , et al., “Modular DNA‐Origami‐Based Nanoarrays Enhance Cell Binding Affinity through the “Lock‐and‐key” Interaction,” Journal of the American Chemical Society 145 (2023): 5447–5455, 10.1021/jacs.2c13825. 36812464

[60] M. Wang , D. L. Yang , Q. Lu , et al., “Spatially Reprogramed Receptor Organization to Switch Cell Behavior Using a DNA Origami‐Templated Aptamer Nanoarray,” Nano Letters 22 (2022): 8445–8454, 10.1021/acs.nanolett.2c02489. 36255126

[61] I. R. Krauss , A. Pica , A. Merlino , L. Mazzarella , and F. Sica , “Duplex–Quadruplex Motifs in a Peculiar Structural Organization Cooperatively Contribute to Thrombin Binding of a DNA Aptamer,” Acta Crystallographica Section D‐Biological Crystallography 69 (2013): 2403–2411, 10.1107/S0907444913022269. 24311581

[62] I. R. Krauss , V. Spiridonova , A. Pica , V. Napolitano , and F. Sica , “Different Duplex/Quadruplex Junctions Determine the Properties of Anti‐Thrombin Aptamers With Mixed Folding,” Nucleic Acids Research 44 (2016): 983–991. 26673709 10.1093/nar/gkv1384 PMC4737158

[63] X. L. Hu , L. L. Tang , M. X. Zheng , et al., “Structure‐Guided Designing Pre‐Organization in Bivalent Aptamers,” Journal of the American Chemical Society 144 (2022): 4507–4514, 10.1021/jacs.1c12593. 35245025

[64] D. S. Goodsell and A. J. Olson , “Structural Symmetry and Protein Function,” Annual Review of Biophysics and Biomolecular Structure 29 (2000): 105–153, 10.1146/annurev.biophys.29.1.105. 10940245

[65] Y. W. Cheung , J. Kwok , A. W. L. Law , R. M. Watt , M. Kotaka , and J. A. Tanner , “Structural Basis for Discriminatory Recognition of Plasmodium Lactate Dehydrogenase by a DNA Aptamer,” Proceedings of the National Academy of Sciences of the United States of America 110 (2013): 15967–15972, 10.1073/pnas.1309538110. 24043813 PMC3791781

[66] S. J. Klug and M. Famulok , “All You Wanted to Know About SELEX,” Molecular Biology Reports 20 (1994): 97–107, 10.1007/BF00996358. 7536299

[67] C. Zhu , Z. R. Feng , H. W. Qin , et al., “Recent Progress of SELEX Methods for Screening Nucleic Acid Aptamers,” Talanta 266 (2024): 124998, 10.1016/j.talanta.2023.124998. 37527564

