# Supplementary material for: Assembly of Protein‐DNA Framework Nanostructures: Structurally Defining Protein‐DNA Interfaces With Aptamer
Source: Angew Chem Int Ed Engl. 2026 Jun 4;65(32):e9624043. doi: 10.1002/anie.9624043 (PMC13427169; doi:10.1002/anie.9624043)
Supplement: Supplementary file 1 — Supporting Information: anie72825‐sup‐0001‐SuppMat.docx. Figure S1: List of the secondary structures of all thrombin bApts. Figure S2: AF3‐aided modeling process. Figure S3: AF3‐aided prediction of oligomeric structures. Figure S4: Images of oligomeric structures. Figure S5: Double‐connected PDF 1D arrays assembled from D17‐m‐10‐tcga (m = 6, 7) and T. Figure S6: Double‐connected PDF 1D ladders assembled from D17‐m‐10‐cg (m = 8–10) and T. Figure S7: Double‐connected PDF 1D arrays assembled from D17‐m‐k‐se (m = 5 or 11; k = 10 or 21) and T. Figure S8: AFM images for co‐assembly of T and D17‐5‐10‐tcga (a) and D17‐11‐10‐cg (b) at different concentrations. Figure S9: AFM images of assemblies formed by T with random sequence (RD‐10‐tcga) and mutant sequence (MD‐5‐10‐tcga), and the assembly of D17‐5‐10‐tcga with FIXa. Figure S10: 2D arrays assembled from D22‐11‐k‐cg (k = 10 or 21) and T. Figure S11: AFM images for co‐assembly of T and D22‐11‐10‐cg at different concentrations. Figure S12: nPAGE (4%) characterization of the trigonal PDF prisms assembled from D17‐m‐2t‐10a/10b (m = 6–11) and T. Figure S13: Cryo‐EM data processing workflow for the D17‐6‐2t‐10a/10b‐T. Figure S14: Cryo‐EM data processing workflow for the D17‐11‐2t‐10a/10b‐T. Figure S15: Workflow for constructing the RE31‐T‐HD22‐based trigonal PDF prisms models fitted into the cryo‐EM density maps. Figure S16: Map of the recombinant plasmid encoding pET28a‐PfLDH. Figure S17: SDS‐PAGE analysis of the PfLDH protein. Figure S18: nPAGE (4%) characterization of the PDF chains co‐assembled by PfLDH and 2008s‐x (x = 15 or 25). Figure S19: PDF chains co‐assembled by PfLDH and its aptamer 2008s‐x. Table S1: Cryo‐EM Data collection parameters of D17‐6‐2t‐10a/10b‐T. Table S2: Cryo‐EM Data collection parameters of D17‐11‐2t‐10a/10b‐T. [file ANIE-65-e9624043-s001.docx]

**Supporting Information**

Assembly of Protein-DNA Framework Nanostructures: Structurally Defining Protein-DNA Interfaces with Aptamers

Zhe Zhang,^⊥,[a,b]^ Xuanyu Nan,^⊥,[c,d]^ Zhengyu Huang,^[a]^ Jin Jin,^[a]^ Cheng Tian,^[a]^ Lian Chen,^[a]^ Xiaoli Hu,^[a]^ Huawei He, *^[e]^ Yuhe Renee Yang,*^[c,f]^ Cheng Zhi Huang,^[a]^ Chengde Mao,*^[g]^ Hua Zuo,*^[a]^

[a] Key Laboratory of Luminescence Analysis and Molecular Sensing (Southwest University), Ministry of Education, College of Pharmaceutical Sciences, Southwest University, Chongqing 400715, China

[b] Yibin Academy of Southwest University, Yibin 644000, China

[c] Laboratory of Nanosystem and Hierarchical Fabrication, National Center for Nanoscience and Technology, Beijing 100190, China

[d] State Key Laboratory of Chemical Resource Engineering, Beijing University of Chemical Technology, Beijing 100029, China

[e] Integrative Science Center of Germplasm Creation in Western China (Chongqing) Science City, Biological Science Research Center, Southwest University, Chongqing, 400715, China

[f] University of Chinese Academy of Sciences, Beijing 100049, China

[g] Department of Chemistry, Purdue University, West Lafayette 47907, IN 47907, USA

*Corresponding author. Email: [zuohua@swu.edu.cn](mailto:zuohua@swu.edu.cn) (H. Zuo), [mao@purdue.edu](mailto:mao@purdue.edu) (C. D. Mao), [yangyh@nanoctr.cn](mailto:yangyh@nanoctr.cn) (Y. R. Yang); [hehuawei@swu.edu.cn](mailto:hehuawei@swu.edu.cn) (H. W. He)

^⊥^These authors contributed equally to this work.

**Table of contents**

[Materials and Methods 3](#_Toc226413854)

[Materials 3](#_Toc226413855)

[Methods 6](#_Toc226413856)

[Supplementary Figures, Tables and Text 11](#_Toc226413857)

[Figure S1 14](#_Toc226413858)

[Figure S2 15](#_Toc226413859)

[Figure S3 16](#_Toc226413860)

[Figure S4 20](#_Toc226413861)

[Figure S5 22](#_Toc226413862)

[Figure S6 25](#_Toc226413863)

[Figure S7 27](#_Toc226413864)

[Figure S8 29](#_Toc226413865)

[Figure S9 30](#_Toc226413866)

[Figure S10 32](#_Toc226413867)

[Figure S11 33](#_Toc226413868)

[Figure S12 34](#_Toc226413869)

[Figure S13 35](#_Toc226413870)

[Figure S14 36](#_Toc226413871)

[Figure S15 37](#_Toc226413872)

[Figure S16 38](#_Toc226413873)

[Figure S17 39](#_Toc226413874)

[Figure S18 39](#_Toc226413875)

[Figure S19 40](#_Toc226413876)

[Table S1 41](#_Toc226413877)

[Table S2 41](#_Toc226413878)

[References 42](#_Toc226413879)

# Materials and Methods

## Materials

Stains-all (C_30_H_27_BrN_2_S_2_), Bradford reagent and ultra centrifugal filters (30 kDa MWCO) were purchased from Sigma-Aldrich (USA). Urea, boric acid, Tris base, EDTA**^.^**Na_2_**^.^**H_2_O, magnesium acetate, acrylamide, bisacylamide, imidazole, Tris-glycine-SDS running buffer, isopropyl β-D-1-thiogalactopyranoside (IPTG) and Coomassie Brilliant Blue R-250 were purchased from Sangon Biotech Co., Ltd. (Shanghai, China). *N,N,N',N'*-Tetramethyl ethylenediamine (TEMED) and ammonium persulfate were purchased from Beijing Dingguo Changsheng Biotech Co., Ltd. (Beijing, China). Acetic acid, hydrochloric acid, absolute ethanol, potassium chloride and sodium chloride were purchased from Chengdu Kelong Chemical Co., Ltd. (Chengdu, China). Ni-NTA resin and yeast extract were purchased from Beijing Solarbio Technology Co., Ltd. (Beijing, China). Tryptone was purchased from Shanghai Aladdin Biochemical Technology Co., Ltd. (Shanghai, China). Kanamycin was purchased from Beyotime Biotech Inc. (Shanghai, China). Human α-thrombin (8.9 mg/mL, MW = 36700) was purchased from Haematologic Technologies Inc. (USA). Human coagulation factor IX (FIXa) (1080) (0.5 mg, MW = 45000) was purchased from Enzyme Research Laboratories Inc. (USA). Highest Grade V1 Mica and Polylysine (0.1 % (w/v), MW = 150,000–300,000) were purchased from Ted Pella, Inc. (USA). Highest Grade V1 Mica and Polylysine (0.1 % (w/v), MW = 150,000–300,000) were purchased from Ted Pella, Inc. (USA). EM grids (R 1.2/1.3 on 300 copper mesh) and Cu grids (200 mesh with reinforced carbon support film) were purchased from Zhongjingkeyi Films Technology Co., Ltd. (Beijing, China). Uranyl formate was purchased from Electron Microscopy Sciences, Inc. (USA). SDS-PAGE sample loading buffer was purchased from Shanghai Epizyme Biomedical Technology Co., Ltd. (Shanghai, China). Deionized (DI) water (resistivity ~ 18.25 MΩ) was used in all experiments.

**Oligonucleotides** were purchased (with ULTRA-PAGE purification) from Sangon Biotech Co., Ltd. (Shanghai, China) and were directly used without further purification. Oligonucleotides sequences are listed below:

**Dn:**

| D13 | CACAGTCcgtGGTAGGGCAGGTTGGggtGACTGTGGGTGCGTAGGTTGGTGTGGTTGGGGCGCACC |
| --- | --- |
| D14 | CACAGTCcgtGGTAGGGCAGGTTGGggtGACTGTGGGGTGCGTAGGTTGGTGTGGTTGGGGCGCACCC |
| D15 | CATCAGTCcgtGGTAGGGCAGGTTGGggtGACTGATGGGGTGCGTAGGTTGGTGTGGTTGGGGCGCACCC |
| D16 | CATCTAGTCcgtGGTAGGGCAGGTTGGggtGACTAGATGGGGTGCGTAGGTTGGTGTGGTTGGGGCGCACCC |
| D17 | CTCATCTAAGTCcgtGGTAGGGCAGGTTGGggtGACTTAGATGAGGTGCGTAGGTTGGTGTGGTTGGGGCGCAC |
| D18 | CATCTAAGTCcgtGGTAGGGCAGGTTGGggtGACTTAGATGAGGGTGCGTAGGTTGGTGTGGTTGGGGCGCACCCT |
| D19 | CATCTAAGTCcgtGGTAGGGCAGGTTGGggtGACTTAGATGGGCAGTGCGTAGGTTGGTGTGGTTGGGGCGCACTGCC |
| D20 | CATCTAAGTCcgtGGTAGGGCAGGTTGGggtGACTTAGATGGGCACGTGCGTAGGTTGGTGTGGTTGGGGCGCACGTGCC |
| D21 | CATCTAAGTCcgtGGTAGGGCAGGTTGGggtGACTTAGATGGGCACGGTGCGTAGGTTGGTGTGGTTGGGGCGCACCGTGCC |
| D22 | CATCTAAGTCcgtGGTAGGGCAGGTTGGggtGACTTAGATGGGCACGTGTGCGTAGGTTGGTGTGGTTGGGGCGCACACGTGCC |
| D23 | CATCTAAGTCcgtGGTAGGGCAGGTTGGggtGACTTAGATGAGGCACGTGTGCGTAGGTTGGTGTGGTTGGGGCGCACACGTGCCT |

**D17-m-k:**

| D17-5-10 | TCTCCGTGAACTCATCTAAGTCcgtGGTAGGGCAGGTTGGggtGACTTAGATGAGGTGCGTAGGTTGGTGTGGTTGGGGCGCAC |
| --- | --- |
| D17-6-10 | TCTCCGTGAATCATCTAAGTCcgtGGTAGGGCAGGTTGGggtGACTTAGATGAGGTGCGTAGGTTGGTGTGGTTGGGGCGCACC |
| D17-7-10 | TCTCCGTGAACATCTAAGTCcgtGGTAGGGCAGGTTGGggtGACTTAGATGAGGTGCGTAGGTTGGTGTGGTTGGGGCGCACCT |
| D17-8-10 | TCTCCGTGAAATCTAAGTCcgtGGTAGGGCAGGTTGGggtGACTTAGATGAGGTGCGTAGGTTGGTGTGGTTGGGGCGCACCTC |
| D17-9-10 | TCTCCGTGAATCTAAGTCcgtGGTAGGGCAGGTTGGggtGACTTAGATGAGGTGCGTAGGTTGGTGTGGTTGGGGCGCACCTCA |
| D17-10-10 | TCTCCGTGAACTAAGTCcgtGGTAGGGCAGGTTGGggtGACTTAGATGAGGTGCGTAGGTTGGTGTGGTTGGGGCGCACCTCAT |
| D17-11-10 | TCTCCGTGAATAAGTCcgtGGTAGGGCAGGTTGGggtGACTTAGATGAGGTGCGTAGGTTGGTGTGGTTGGGGCGCACCTCATC |
| D22-11-10 | TCTCCGTGAATACTGTAAGTCcgtGGTAGGGCAGGTTGGggtGACTTACAGTAGATGAGGTGCGTAGGTTGGTGTGGTTGGGGCGCACCTCATC |
| D17-5-21 | TCTCACATTGGTCTCCGTGAACTCATCTAAGTCcgtGGTAGGGCAGGTTGGggtGACTTAGATGAGGTGCGTAGGTTGGTGTGGTTGGGGCGCAC |
| D17-11-21 | TCTCACATTGGTCTCCGTGAATAAGTCcgtGGTAGGGCAGGTTGGggtGACTTAGATGAGGTGCGTAGGTTGGTGTGGTTGGGGCGCACCTCATC |
| D22-11-21 | TCTGACATTGGTCTCCGTGAATACTGTAAGTCcgtGGTAGGGCAGGTTGGggtGACTTACAGTAGATGAGGTGCGTAGGTTGGTGTGGTTGGGGCGCACCTCATC |

**Control strands** **(Black text indicates sequences mutated from D17-5-10):**

| RD-10 | TCTCCGTGAAGCATCCGAGTTAGTCGTACGCTAAGCGTATTGACCGTATCGATGAGCTAGCTTACCGTATACGGCATCGACGTA |
| --- | --- |
| MD-5-10 | TCTCCGTGAACTCATCTAAGTCcgtCATACTGCAACTTATggtGACTTAGATGAGGTGCGTACTTTCTGTACTTGGGGCGCAC |

**17-m-2t-10a/17-m-2t-10b:**

| 17-6-2t-10a | TCTCCGTGAAttTCATCTAAGTCcgtGGTAGGGCAGGTTGGggtGACTTAGATGAGGTGCGTAGGTTGGTGTGGTTGGGGCGCACC |
| --- | --- |
| 17-6-2t-10b | TTCACGGAGAttTCATCTAAGTCcgtGGTAGGGCAGGTTGGggtGACTTAGATGAGGTGCGTAGGTTGGTGTGGTTGGGGCGCACC |
| 17-7-2t-10a | TCTCCGTGAAttCATCTAAGTCcgtGGTAGGGCAGGTTGGggtGACTTAGATGAGGTGCGTAGGTTGGTGTGGTTGGGGCGCACCT |
| 17-7-2t-10b | TTCACGGAGAttCATCTAAGTCcgtGGTAGGGCAGGTTGGggtGACTTAGATGAGGTGCGTAGGTTGGTGTGGTTGGGGCGCACCT |
| 17-8-2t-10a | TCTCCGTGAAttATCTAAGTCcgtGGTAGGGCAGGTTGGggtGACTTAGATGAGGTGCGTAGGTTGGTGTGGTTGGGGCGCACCTC |
| 17-8-2t-10b | TTCACGGAGAttATCTAAGTCcgtGGTAGGGCAGGTTGGggtGACTTAGATGAGGTGCGTAGGTTGGTGTGGTTGGGGCGCACCTC |
| 17-9-2t-10a | TCTCCGTGAAttTCTAAGTCcgtGGTAGGGCAGGTTGGggtGACTTAGATGAGGTGCGTAGGTTGGTGTGGTTGGGGCGCACCTCA |
| 17-9-2t-10b | TTCACGGAGAttTCTAAGTCcgtGGTAGGGCAGGTTGGggtGACTTAGATGAGGTGCGTAGGTTGGTGTGGTTGGGGCGCACCTCA |
| 17-10-2t-10a | TCTCCGTGAAttCTAAGTCcgtGGTAGGGCAGGTTGGggtGACTTAGATGAGGTGCGTAGGTTGGTGTGGTTGGGGCGCACCTCAT |
| 17-10-2t-10b | TTCACGGAGAttCTAAGTCcgtGGTAGGGCAGGTTGGggtGACTTAGATGAGGTGCGTAGGTTGGTGTGGTTGGGGCGCACCTCAT |
| 17-11-2t-10a | TCTCCGTGAAttTAAGTCcgtGGTAGGGCAGGTTGGggtGACTTAGATGAGGTGCGTAGGTTGGTGTGGTTGGGGCGCACCTCATC |
| 17-11-2t-10b | TTCACGGAGAttTAAGTCcgtGGTAGGGCAGGTTGGggtGACTTAGATGAGGTGCGTAGGTTGGTGTGGTTGGGGCGCACCTCATC |

**k-se:**

| 10-cg | TTCACGGAGAcg |
| --- | --- |
| 10-tcga | TTCACGGAGAtcga |
| 21-cg | TTCACGGAGACCAATGTGAGAcg |
| 21-tcga | TTCACGGAGACCAATGTGAGAtcga |

**2008s-x:**

| 2008s-15 | TCAGTGACTACTATCGACTCCCTGGGCGGTAGAACCATAGTGACCCAGCACTGAGGAGTCGATAGTAGT |
| --- | --- |
| 2008s-25 | TCAGTGCAATGCCTGCACTACTATCGACTCCCTGGGCGGTAGAACCATAGTGACCCAGCACTGAGGAGTCGATAGTAGTGCAGGCATTG |

(The color coding in the sequence is following. green: duplex linker in a bApt; red: aptamer RE31; purple: aptamer HD22; blue: side branch in a bApt; orange: TT spacers; magenta: sticky ends)

## Methods

**nPAGE.** Native PAGE that contained 4% polyacrylamide (acrylamide/bisacrylamide 19:1 (40%, w/v)) was run on a SE 600 electrophoresis system (Hoefer, Inc.) at 25 ℃ (220 V, constant voltage). Each lane contained 1 μg DNA sample. TAE/Mg^2+^/K^+^ buffer (40 mM Tris base, 20 mM acetic acid, 2 mM EDTA**^.^**Na_2_**^.^**H_2_O, 12.5 mM magnesium acetate and 10 mM potassium chloride, pH 7.4) was used both as the running buffer and the buffer in the gel. After electrophoresis, the gels were stained with Stains-All and scanned with a LIDE 120 scanner (Canon, Inc.). The band intensities in the gels were measured using Image *J*, a computer software for image processing ^[1]^. The yield of PDFs was calculated as the ratio of the band intensity corresponding to the target structure to the total intensity of all bands within the same lane.

**Sodium dodec****yl sulfate-polyacrylamide gel electrophoresis (SDS-PAGE).** SDS-PAGE was performed using a 15% separating gel (15% (w/v) acrylamide, 0.1% SDS (w/v) and 375 mM Tris-HCl, pH 8.8.) and a 5% stacking gel (15% (w/v) acrylamide, 0.1% SDS (w/v) and 125 mM Tris-HCl, pH 6.8). The stacking gel and resolving gel located in the upper and lower layers, respectively. Protein samples (10–20 μg per lane) were denatured in SDS-PAGE sample loading buffer at 100 ℃ for 10 min. Electrophoresis was carried out on a Mini-PROTEAN tetra vertical electrophoresis system (Bio-Rad Laboratories, Inc.) at room temperature, using Tris-glycine-SDS running buffer (25 mM Tris base, 192 mM glycine and 0.1% (v/v) SDS, pH 8.3), with constant voltage (120 V). Gels were stained with Coomassie Brilliant Blue solution (0.1% (w/v) Coomassie Brilliant Blue R-250, 5% (v/v) absolute ethanol and 10% (v/v) acetic acid), followed by destaining with a bleaching solution (5% (v/v) absolute ethanol and 10% (v/v) acetic acid). The gels were then scanned using a LiDE 120 scanner (Canon, Inc.).

**Assembly of bivalent aptamers (bApts) or 2008s-x.** DNA solutions in TAE/Mg^2+^/K^+^ buffer were thermally annealed at 95 ℃ for 5 min, 65 ℃ for 30 min, 50 ℃ for 30 min, 37 ℃ for 30 min, 25 ℃ for 30 min, and then 4 ℃ for 2 h. *(1)* Dn. Strand Dn at a final concentration of 1.0 μM. *(2)* D17-m-k-se, RD-10-tcga, MD-5-10-tcga and D22-m-k-se. Equimolar mixtures of D17-m-k, RD-10, MD-5-10 or D22-m-k with k-se, each at a final concentration of 1.0 μM. *(3)* D17-m-2t-10a/10b. Equimolar mixture of D17-m-2t-10a and D17-m-2t-10b, each at a final concentration of 10.0 μM. *(4)* 2008s-x. Strand 2008s-x at a final concentration of 2.0 μM.

**Assembly of PDF nanostructures.** *(1)* Assembly of PDF oligomers in solution. Annealed DNA bApts (Dn) and thrombin were mixed at equal molar ratio (final concentration: 200 nM each) in TAE/Mg^2+^/K^+^ buffer and incubated at 25 ℃ for 2 h. *(2)* Assembly of PDF 1D chains/ladders and 2D arrays on mica. bApts D17-m-k-se/RD-10-tcga/MD-5-10-tcga/D22-m-k-se and thrombin/FIXa were mixed at equal molar ratio (final concentration: 62.5 nM, 125 nM, 250 nM, 500 nM or 1000 nM each) in TAE/Mg^2+^/K^+^ buffer and incubated at 25 ℃ for 2 h. Then 8 µL of the mixture solution was deposited onto a freshly cleaved mica (Highest Grade V1 Mica, 25 × 75 mm (1 × 3"), Ted Pella, Inc.) surface. The mica was immediately placed in a sealed TC-treated culture dish (35 mm × 10 mm, Corning, Inc.) with 0.5 mL TAE/Mg^2+^/K^+^ buffer spread along the edge of the dish to control the humidity. The dish was then placed in a sealed and moist plastic polyethylene box. The box was kept at 25 ℃ for 24 h. *(3)* Assembly of PDF trigonal prisms in solution. D17-m-2t-10a/10b and thrombin were mixed at equal molar ratio (final concentration: 4 μM or 8 μM each) in TAE/Mg^2+^/K^+^ buffer and incubated at 25 °C for 2 h. *(4)* Assembly of *Pf*LDH-2008s complexes in solution. 2008s-x and *Pf*LDH were mixed at a molar ratio of 1:2 (final concentration: 500 nM for 2008s and 1000 nM for *Pf*LDH) in TAE/Mg^2+^ buffer (40 mM Tris base, 20 mM acetic acid, 2 mM EDTA**^.^**Na_2_**^.^**H_2_O and 12.5 mM magnesium acetate, pH 7.4) and incubated at 25 ℃ for 2 h.

**AFM imaging.** All the AFM images were captured by ScanAsyst in fluid mode on a Multimode 8 AFM (Bruker) using ScanAsyst-Fluid+ probes (Bruker). The mica surface assembly structures (PDF 1D chains/ladders or 2D arrays) were washed with 25 µL TAE/Mg^2+^/K^+^ buffer for four times and imaged in the same buffer. Fast Fourier transform (FFT) was performed by Spectrum 2D function in the software Nanoscope Analysis 3.00 (Bruker). For the samples assembled in solution, 20 μL 5 μg/mL polylysine (Ted Pella, Inc.) was first deposited on freshly cleaved mica surface for 5 min and blew away by nitrogen. 20 μL H_2_O was added to wash the mica surface and blew away immediately by nitrogen. Then 6 µL solution containing PDF oligomers was deposited onto the polylysine-coated mica surface and incubated for 3 min. Finally, 20 μL of TAE/Mg^2+^/K^+^ buffer (for PDF oligomers) or TAE/Mg^2+^ buffer (for *Pf*LDH-2008s-x complexes) was deposited on the mica surface and scanned in fluid mode.

**Electron microscopy (EM) with negative stain of PDF triangle.** 3.5 µL 200 nM D17-T complex solution in TAE/Mg^2+^/K^+^ buffer was applied to Cu grid (Zhongjingkeyi, 200 mesh with reinforced carbon support film, Beijing Zhongjingkeyi Films Technology Co., Ltd.). After a 1-min deposition, the grid was blotted with filter paper and then stained with 2% (w/v) uranyl formate (Electron Microscopy Sciences, Inc.) for 40 s. Micrographs were acquired using a JEM-2100F high-resolution transmission electron microscope (JEOL, Ltd.) operated at 200 kV, with images recorded at a magnification of ×30,000 using SerialEM for automated data collection. Two-dimensional (2D) class averages were first obtained using Relion 4.0.1 ^[2]^. Selected particles were then transferred to cryoSPARC v4.6.0 ^[3]^ for Ab-Initio Reconstruction without any reference volume (Figure S4). The resulting map was displayed in UCSF ChimeraX ^[4]^.

**Cryo-EM: sample preparation and data collection of trigonal PDF prisms.** 4 μM D17-6-2t-10a/10b-T and 8 μM D17-11-2t-10a/10b-T complexes in TAE/Mg^2+^/K^+^ buffer were used for cryo-EM. Prior to cryo-EM grid preparation, the EM grids (Quantifoil, R 1.2/1.3 on 300 copper mesh, Quantifoil Micro Tools GmbH) were glow-discharged for 20 s using a SuPro Coolglow plasma cleaner (Supro). Then, 4 μL of the complex was deposited onto the grids for 4 s, immediately blotted for 4 s with filter paper under 100% relative humidity at 4 °C, and then plunged into liquid ethane to vitrify the samples using the FEI Vitrobot system (FEI). For D17-6-2t-10a/10b-T, cryo-EM data were collected on an FEI Talos Arctica transmission electron microscope (Thermo Fisher Scientific, 9950610) operating at 200 kV and equipped with a K2 direct electron detector, as well as on an FEI Titan Krios transmission electron microscope (Thermo Fisher Scientific, D3418) operating at 300 kV and equipped with a Gatan K3 Summit direct electron detector and a GIF energy filter. Automated data acquisition was performed using SerialEM software. Data collected on the Talos Arctica were recorded at a nominal magnification of 45,000×, corresponding to a pixel size of 0.94 Å, with a defocus range of −1.2 to −1.5 µm. Data collected on the Titan Krios were recorded at a nominal magnification of 105,000×, corresponding to a pixel size of 0.8374 Å. Images were collected over a defocus range of −1.0 to −1.6 µm. For both datasets, each movie stack was recorded with a total electron dose of 50 e⁻/Å², fractionated into 32 frames. For D17-11-2t-10a/10b-T, cryo-EM data were acquired on an FEI Titan Krios transmission electron microscope (Thermo Fisher Scientific, D3172) operating at 300 kV and equipped with a Gatan K3 Summit direct electron detector. Automated data collection was carried out using SerialEM software at a nominal magnification of 29,000×, corresponding to a pixel size of 0.97 Å, with a defocus range of −1.0 to −1.6 µm. Each movie stack was recorded with a total electron dose of 50 e⁻/Å², fractionated into 32 frames.

**Cryo-EM: image processing.** Cryo-EM micrographs were processed using cryoSPARC v4.6.0 ^[3]^. Contrast transfer function (CTF) parameters were determined using Patch CTF Estimation. Particles were initially manually picked with the Manual Picker, extracted from micrographs, and subjected to reference-free two-dimensional (2D) classification. Based on the resulting 2D class averages, particles were re-picked using the Template Picker, extracted with a 460-pixel box size, and refined through multiple iterative rounds of 2D classification to select high-quality particles. The selected particles were first used for reference-free Ab-initio reconstruction to obtain an initial map. This was followed by iterative refinement processes, including homogeneous refinement, heterogeneous refinement, cryoPROS ^[5]^, and non-uniform refinement. All Cryo-EM visualizations were rendered using ChimeraX ^[4]^. Data collection parameters and the complete processing workflow are summarized in Tables S1 and S2 (Supporting Information) and illustrated in Figures S10 and S11 (Supporting Information), respectively.

**Construction of** **recombinant plasmid encoding *Plasmodium falciparum* lactate dehydrogenase (*Pf*LDH)**. The full-length gene of *Pf*LDH (GenBank: DQ825436.1; Length: 951 bp) was obtained from GenBank. The 6×His-Tag was added to the N-terminal, then cloned to the vector pET-28a(+) (Kanamycin) through the 5’ (NcoI) and 3’ (XhoI) digestion sites, and the recombinant plasmid pET28a-*Pf*LDH (tag at N-terminal) were constructed successfully (Figure S11). The gene synthesis technology was provided by Suzhou Genewiz Biotechnology Co., Ltd.

**Transduction of recombinant plasmid encoding *Plasmodium falciparum* lactate dehydrogenase (*Pf*LDH).** 2 μL plasmid and 50 μL BL21 (DE3) competent cells (Shanghai Sangon Biotech Co., Ltd.) were added to a 1.5 mL Eppendorf tube and gently mixed. The mixture was incubated on ice for 30 min, followed by heat shock at 42 ℃ for 90 s. Then the tube was quickly transferred to ice for 5 min to allow the cells to recover. Subsequently, 500 μL of antibiotic-free Luria-Bertani (LB) medium (1% (w/v) tryptone, 1% (w/v) sodium chloride and 0.5 (w/v) yeast extract) was added, and the culture (bacterial solution) was incubated at 37 °C with shaking at 220 rpm for 30 min to facilitate bacterial recovery.

**Expression and purification of *Plasmodium falciparum* lactate dehydrogenase (*Pf*LDH)**. 100 μL bacterial solution was transferred into 10 mL LB medium (1% (w/v) tryptone, 1% (w/v) sodium chloride, 0.5 (w/v) yeast extract and 30 μg/mL kanamycin) and incubated at 37 ℃ with shaking at 220 rpm overnight. It was then transferred into 1 L fresh LB medium and cultured at 37 ℃ with shaking at 220 rpm until the optical density at 600 nm (OD_600_) reached a value between 0.4 and 0.6, measured using a Synergy H1 Multimode Reader (BioTek Instruments, Inc.). Protein expression was induced by adding 0.5 mM isopropyl β-D-1-thiogalactopyranoside (IPTG; Shanghai Sangon Biotech Co., Ltd., China), followed by incubation at 25 ℃ with shaking at 220 rpm for approximately 10 h. After induction, the culture was centrifuged by a TGL-16K High-speed Refrigerated Centrifuge (Hunan Xiangyi Laboratory Instrument Development Co., Ltd.) at 6000 rpm for 20 min at 4 ℃ to harvest the cells. The resulting pellet was resuspended in Buffer A (50 mM Tris base, 500 mM sodium chloride and 10% (v/v) glycerol, pH 7.4) at a ratio of 1:3 (w/v) and subjected to sonication by a JY92-II Ultrasonic Cell Crusher (Ningbo Scientz Biotechnology Co., Ltd.) in an ice-water bath using a pulse program (1 s on, 3 s off) for a total of 30 min at 40% amplitude. The lysate was centrifuged at 12,000 rpm for 30 min at 4 ℃ to remove cellular debris, and the supernatant was filtered through a Sterile Syringe Filter (Sangon Biotech, 0.45 μm/33 mm (aquo-system), Shanghai Sangon Biotech Co., Ltd.). After filtration, the lysate was applied to Ni-NTA resin (Solarbio, His_6_-Tag, Beijing Solarbio Technology Co., Ltd.) for purification. A 40 mL portion of the supernatant was incubated with 5 mL resin, which was then washed with Buffer B (50 mM Tris base, 50 mM imidazole, 500 mM sodium chloride and 10% (v/v) glycerol, pH 7.4) until no further color change was observed upon addition of Bradford Reagent (Sigma-Aldrich). The color shift from brown to blue, which corresponded to the detection of non-specific proteins, ceased, indicating the complete removal of such proteins. The target protein was subsequently eluted using Buffer C (50 mM Tris base, 200 mM imidazole, 500 mM sodium chloride and 10% (v/v) glycerol, pH 7.4), and the eluate was concentrated to 200 ng/μL using an ultra centrifugal filters (Amicon, 30 kDa MWCO, Merck KGaA).

# Supplementary Figures, Tables and Text


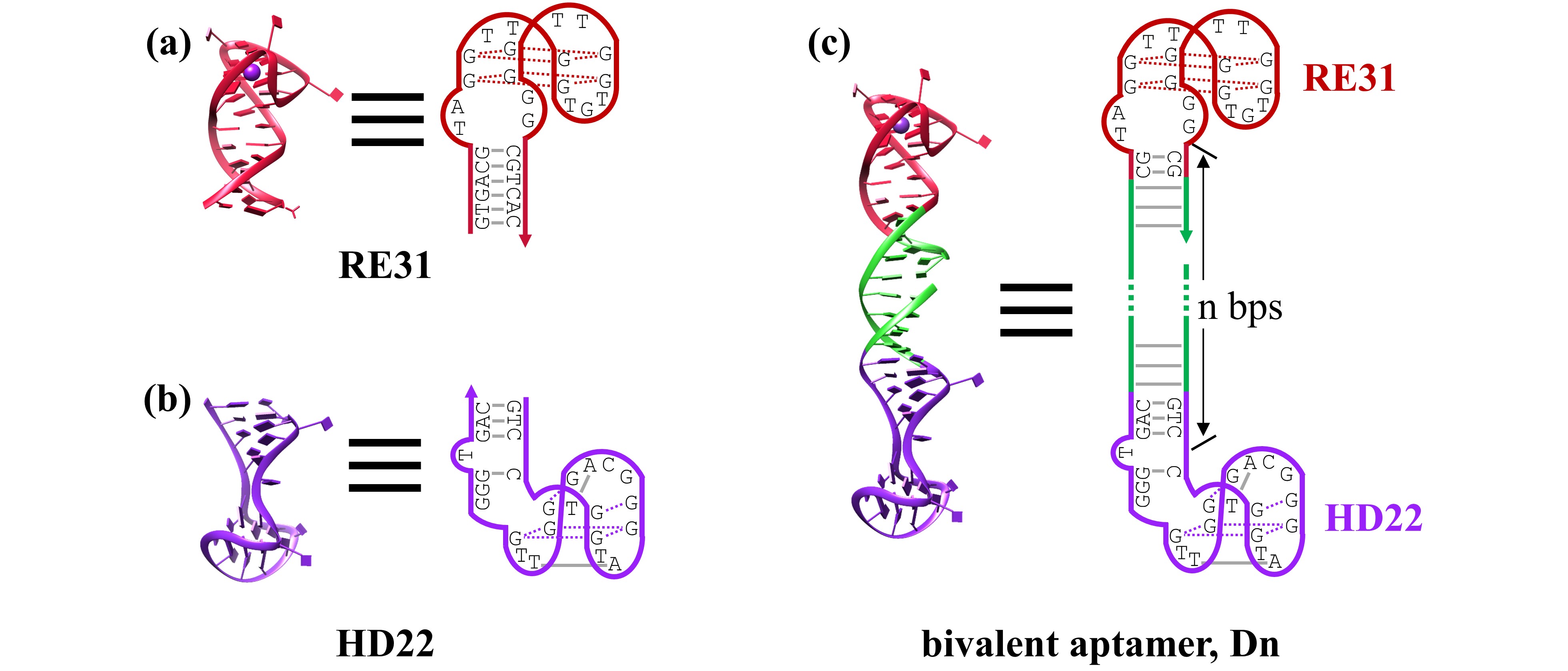


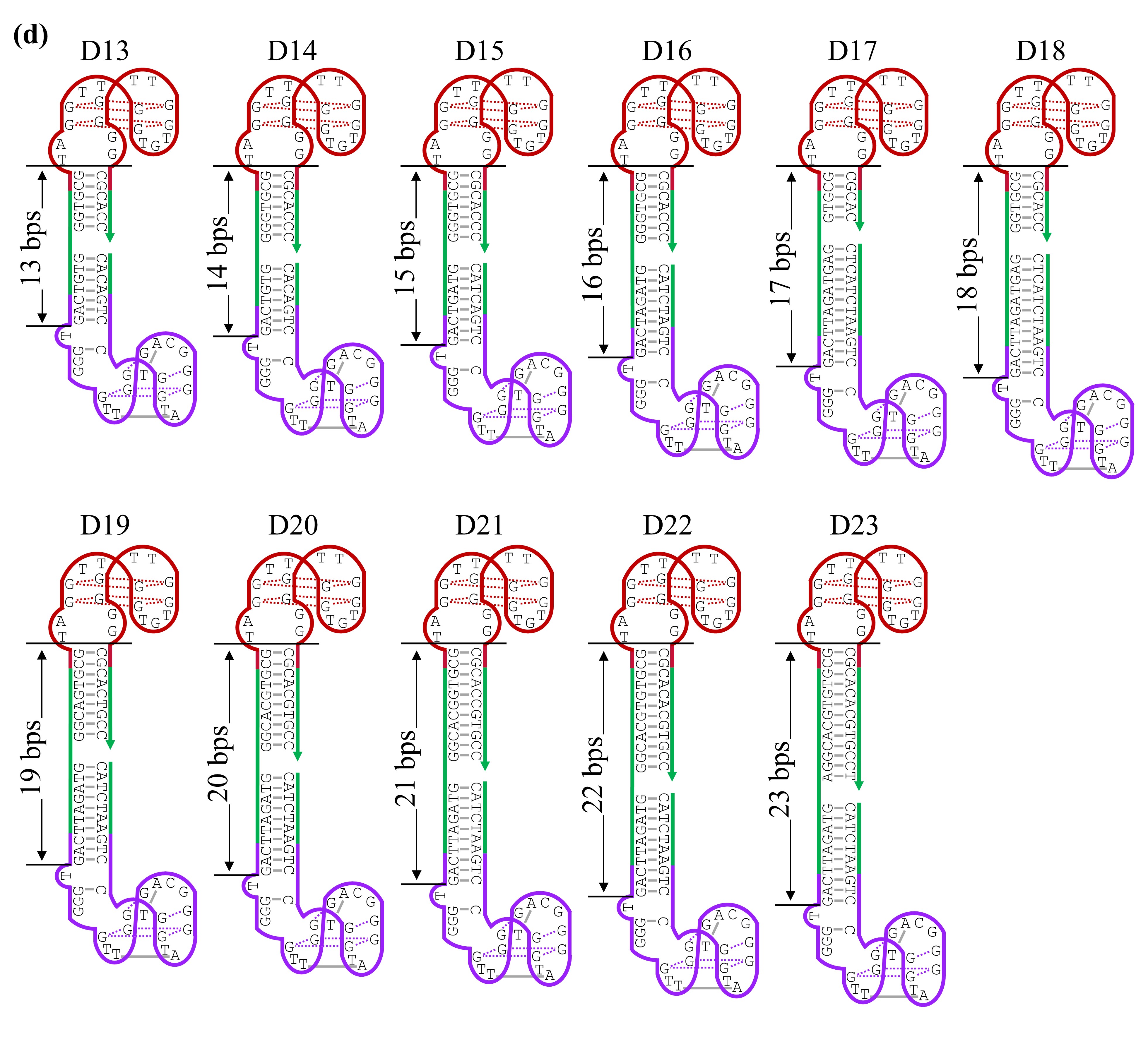


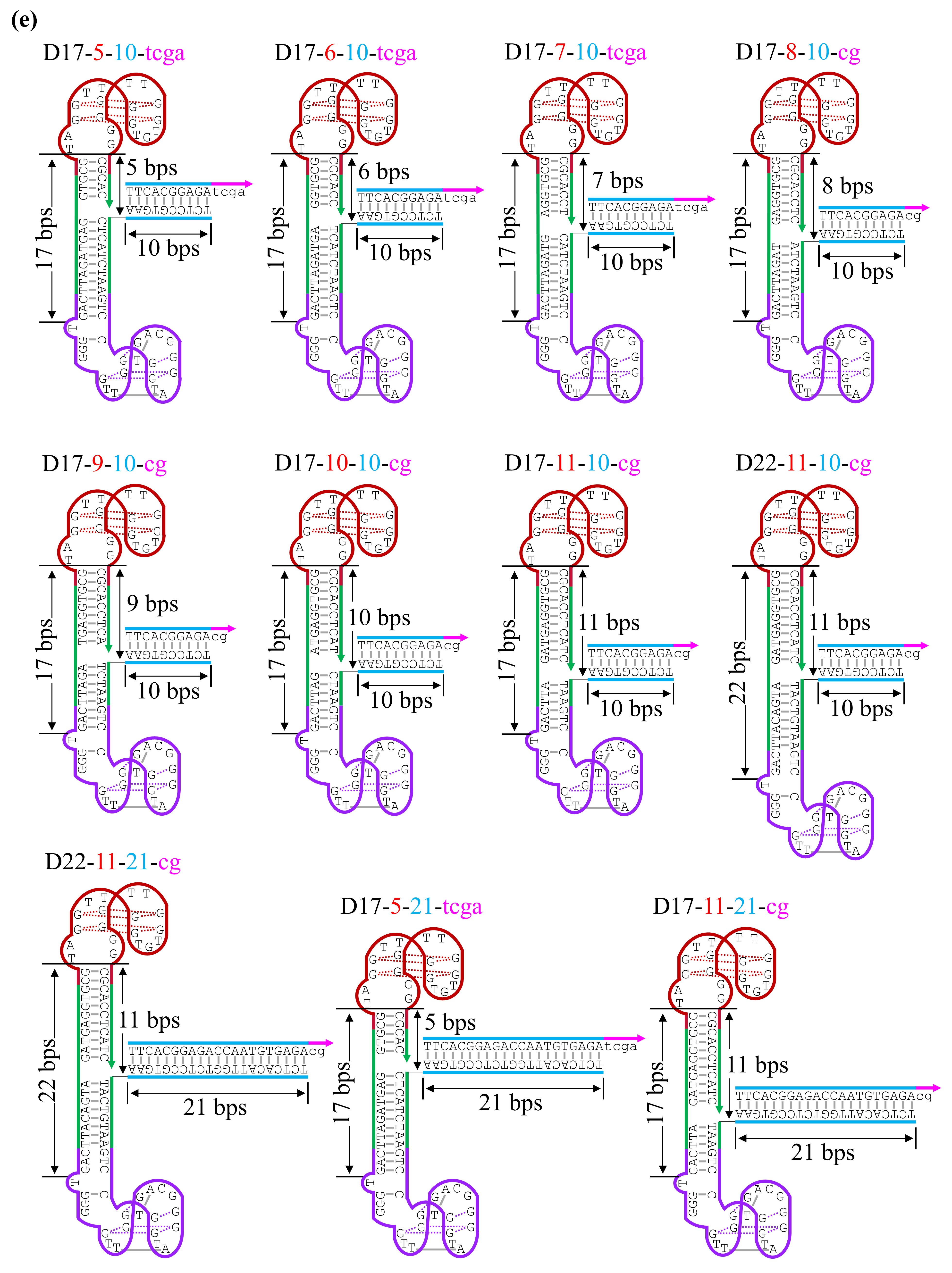


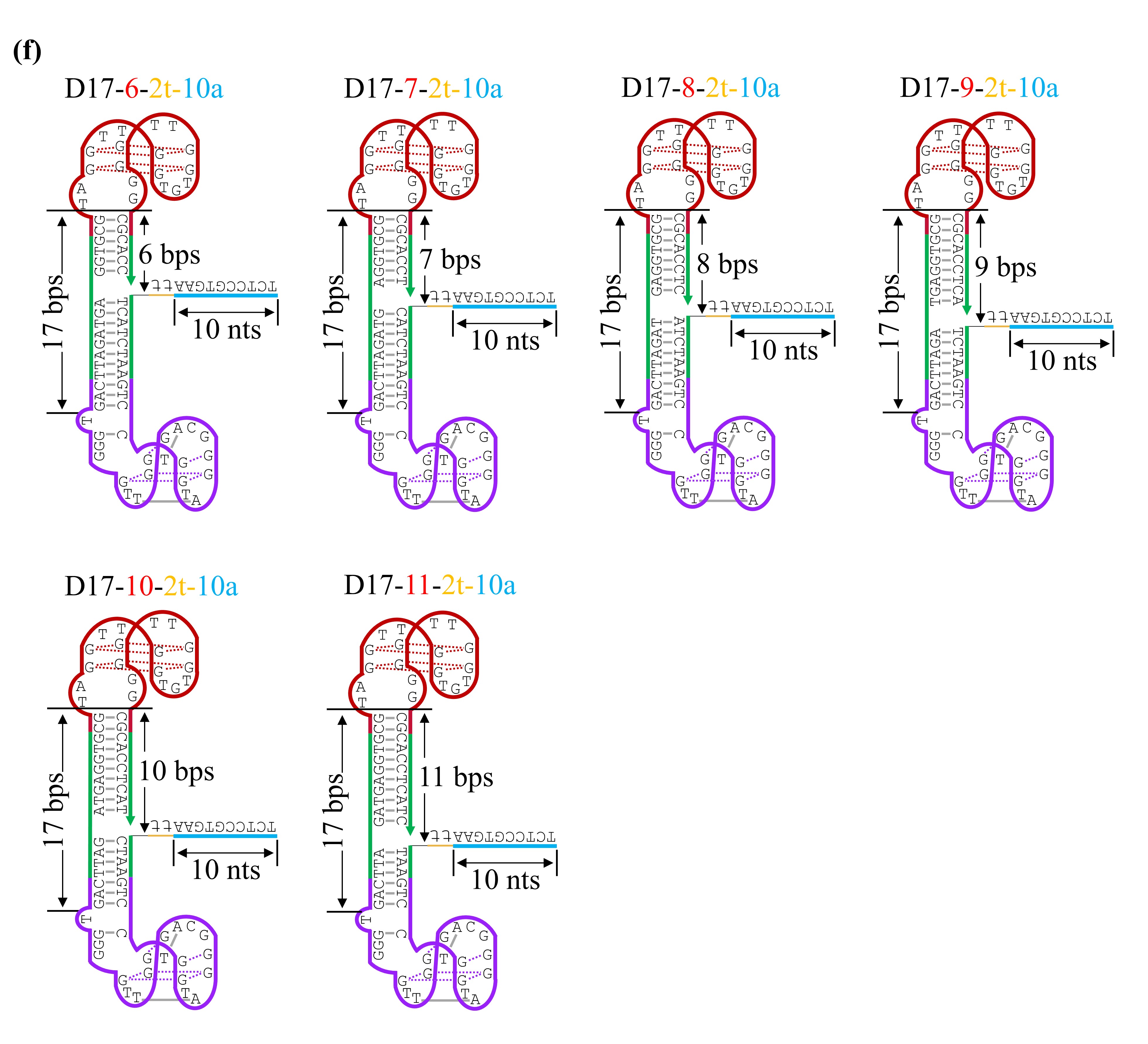


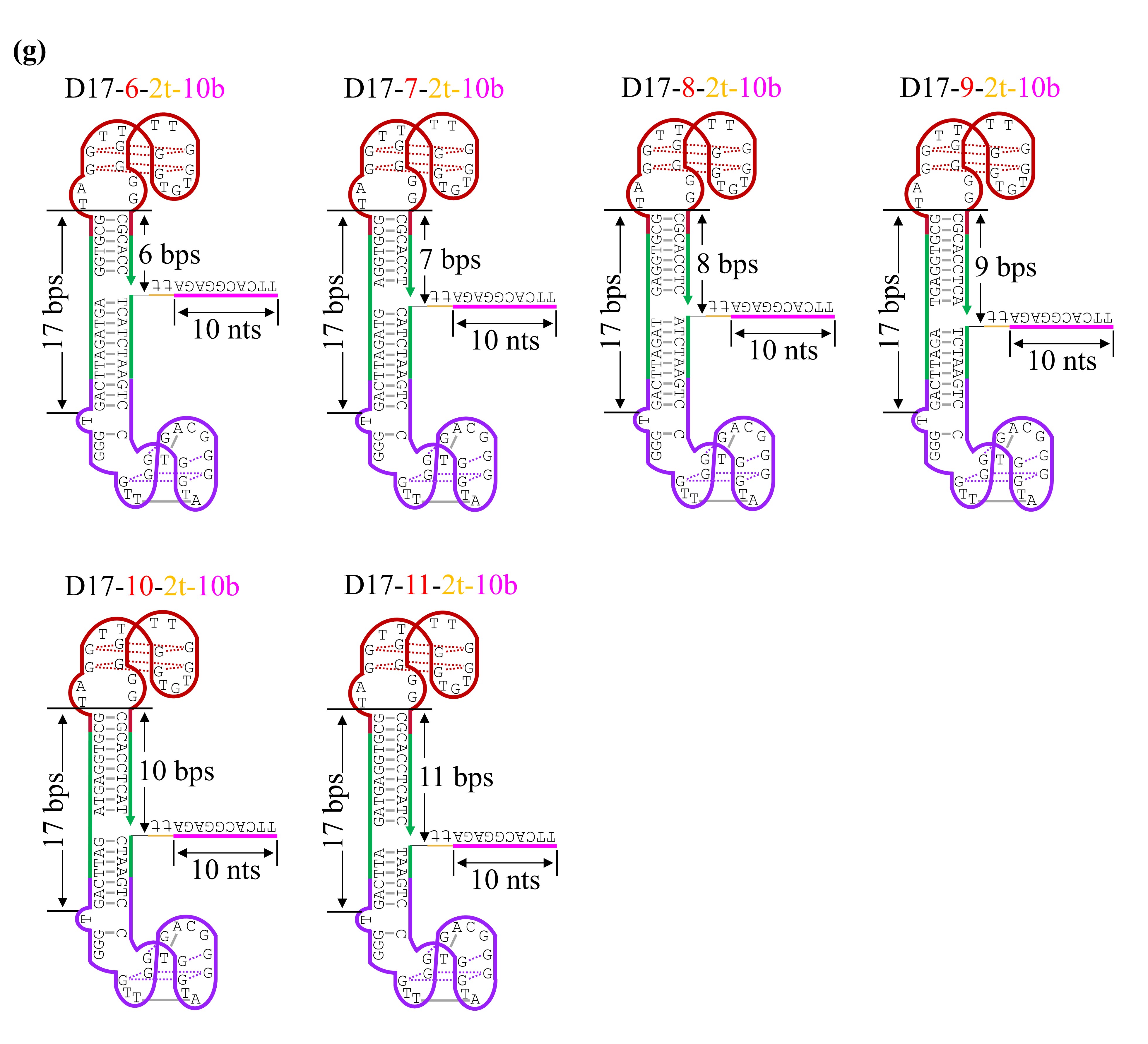


Figure S1**.** List of the secondary structures of all thrombin bApts.


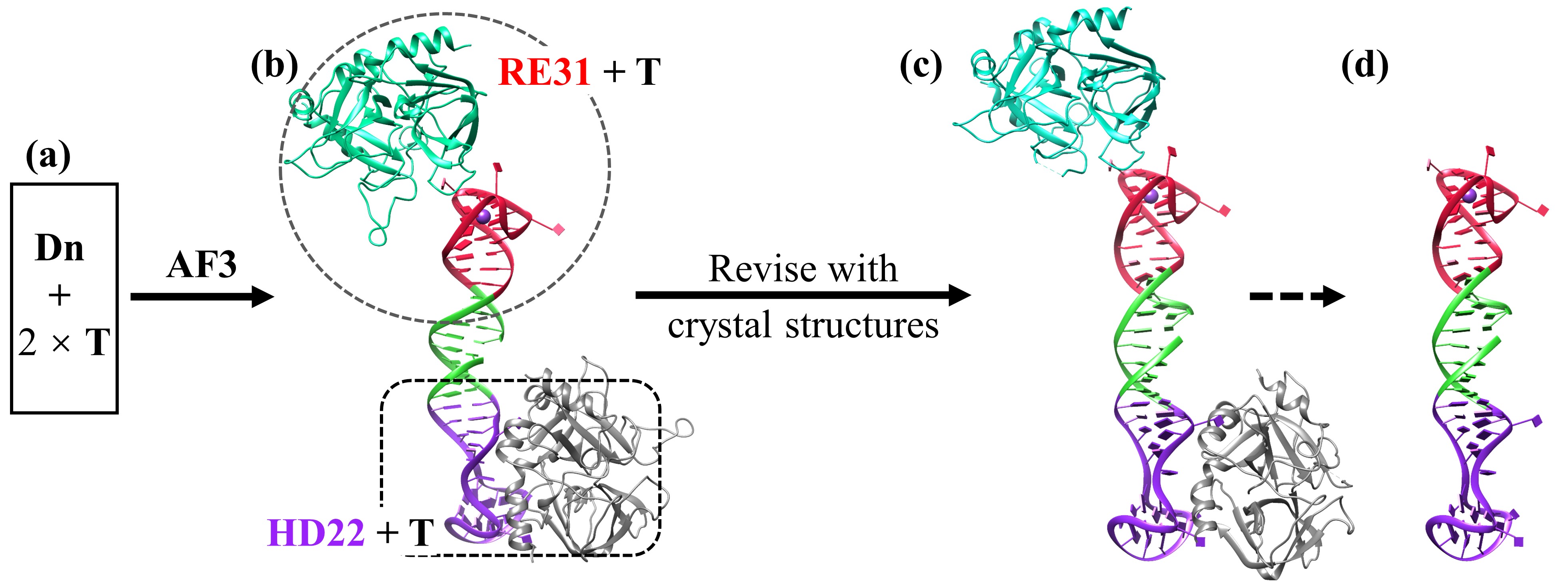


Figure S2**.** AF3-aided modelling process. (a) Input of the sequences of Dn and two thrombin molecules. (b) Structural model of the complex comprising one Dn and two thrombin molecules generated by AF3. (c) The revised Dn-T complex model was constructed by replacing the thrombin-aptamer segments with the corresponding crystal structures. (d) Removal of thrombin molecules from the Dn-T complex yielded the structural model of Dn.


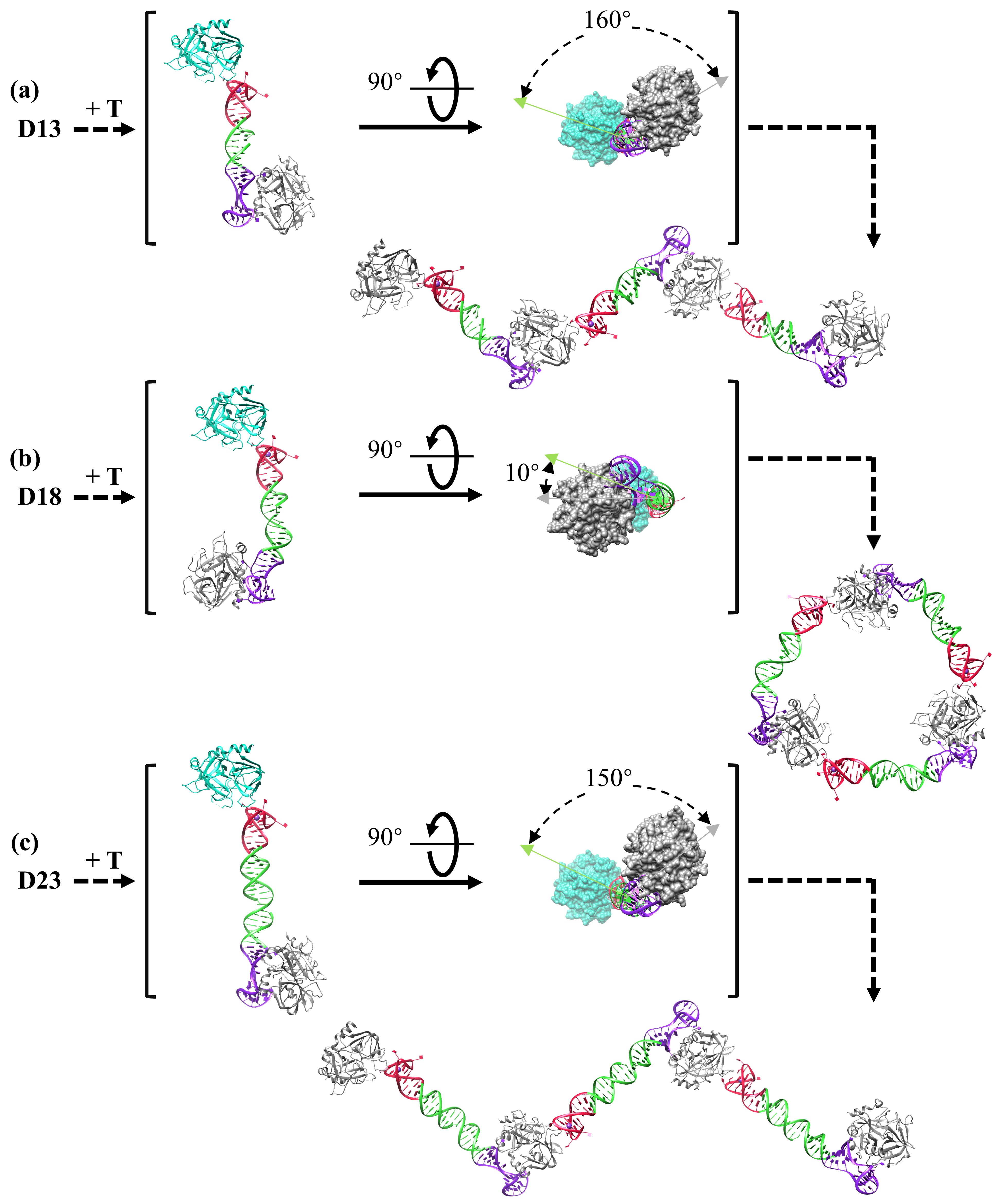


Figure S3**.** AF3-aided prediction of oligomeric structures. (a) Predicted structure of (D13)_1_(T)_2_, showing the dihedral angle (α) of 160° formed between two thrombin molecules in a T-D13-T complex, which ultimately assemble into zig-zag chains. (b) Predicted structure of (D18)_1_(T)_2_, showing the dihedral angle (α) of 10°, which ultimately assemble into cyclotrimer. (c) Predicted structure of (D23)_1_(T)_2_, showing the dihedral angle (α) of 150°, which ultimately assemble into zig-zag chains.


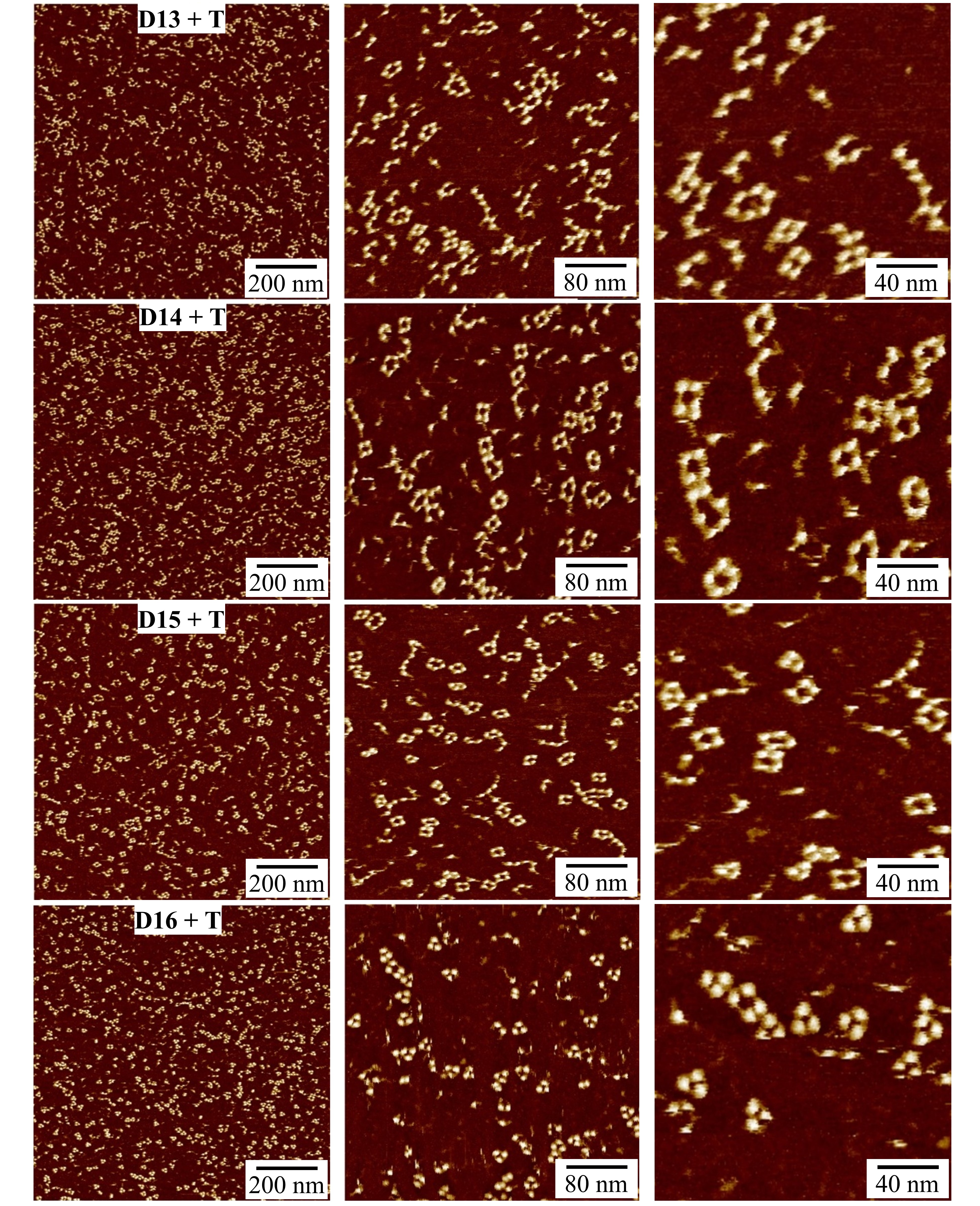

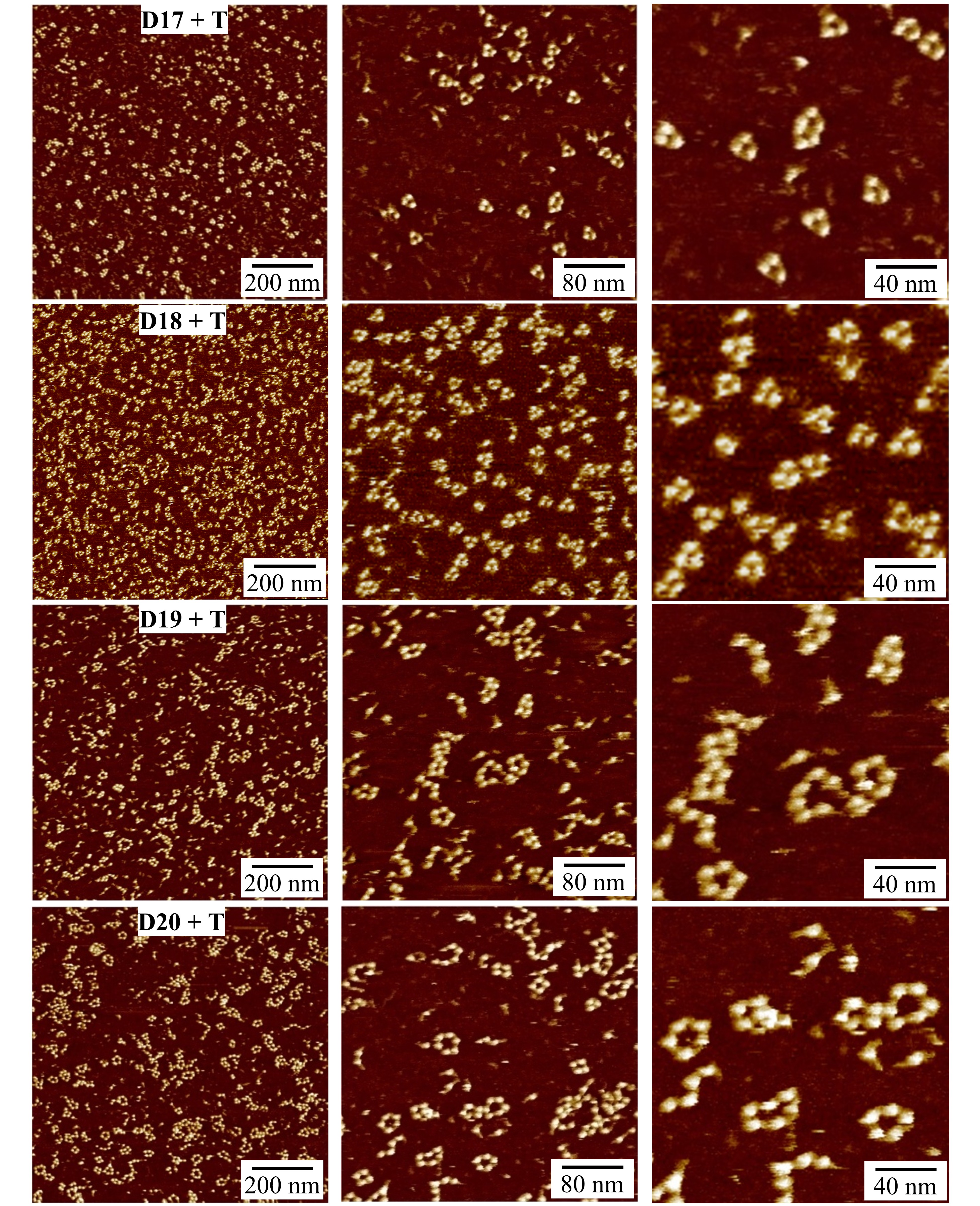

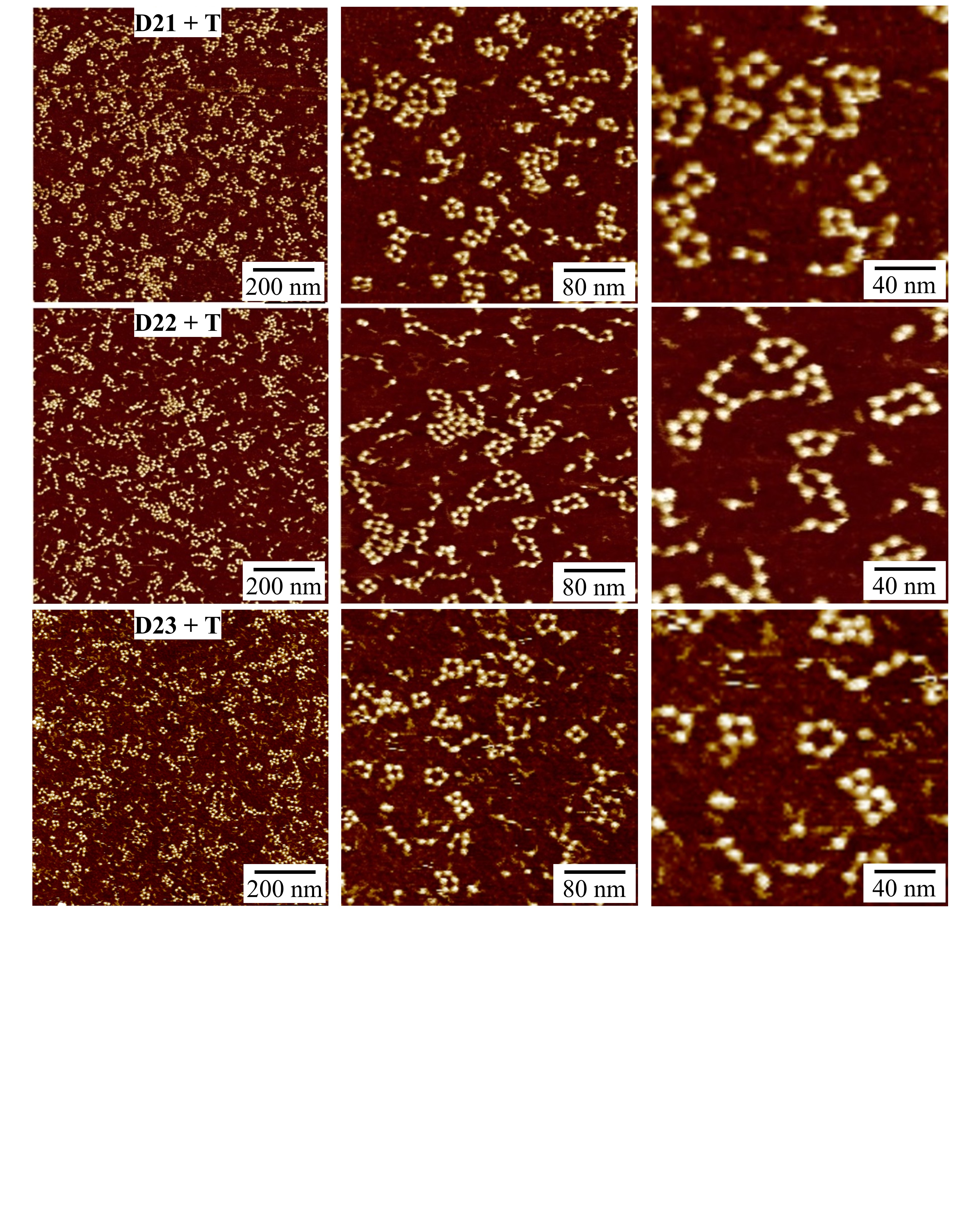





Figure S4**.** Images of oligomeric structures. (a) AFM images of Dn-T complexes. (b) Ns-EM images, representative 2D class and 3D reconstructions of D17-T complex.


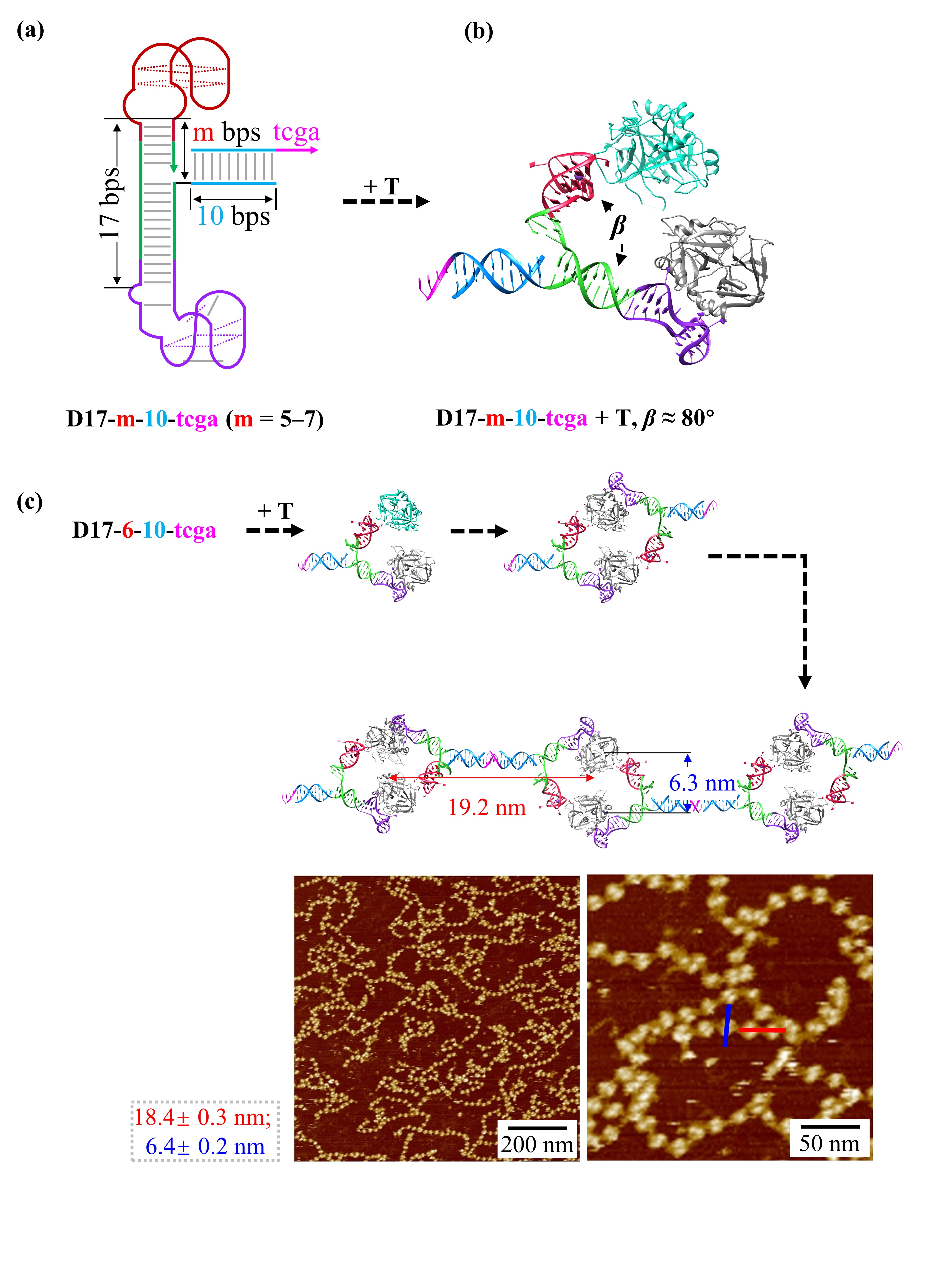

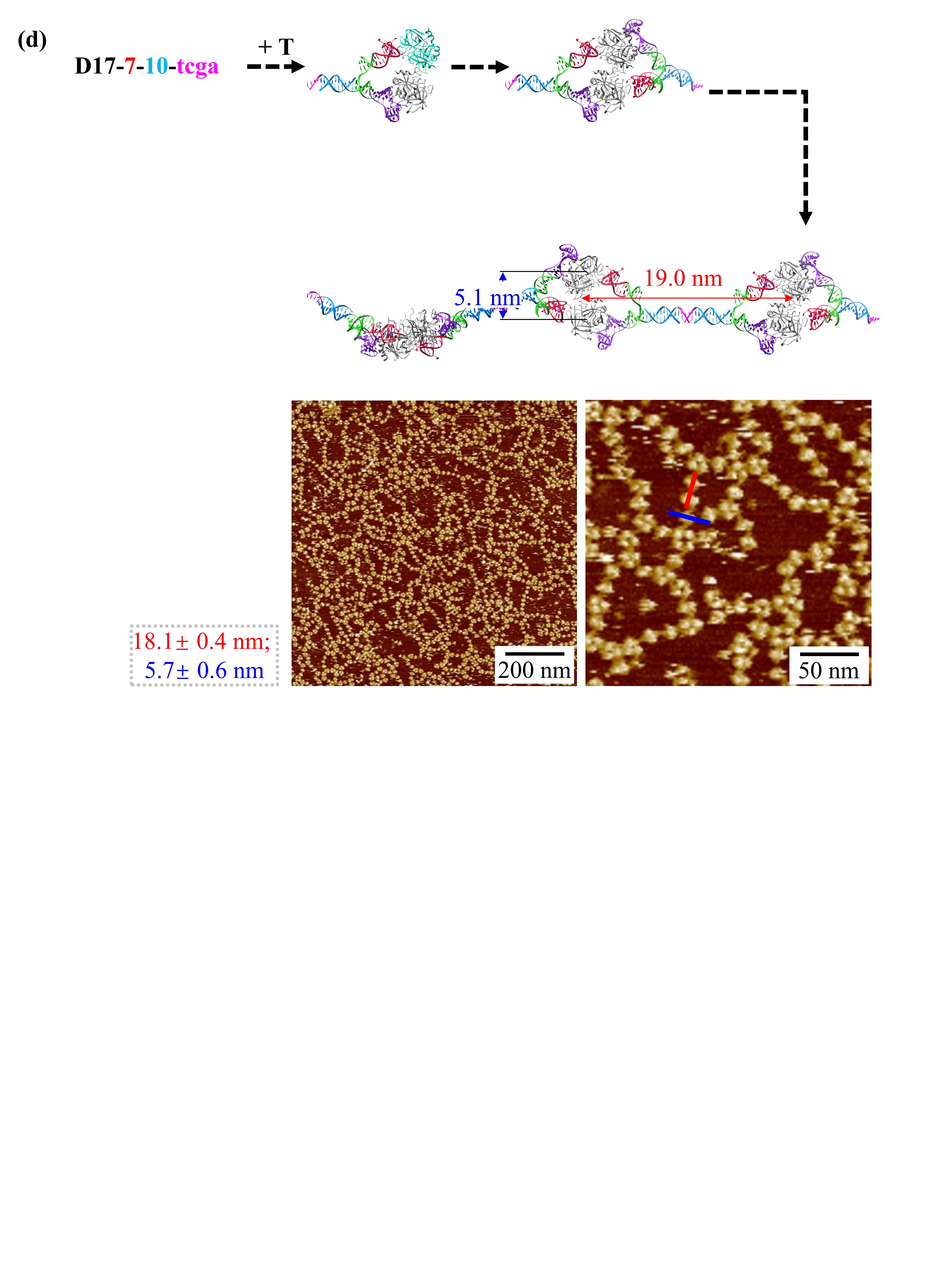


Figure S5**.** Double-connected PDF 1D arrays assembled from D17-m-10-tcga (m = 6, 7) and T. (a) Scheme of the secondary structure of D17-m-10-tcga. (b) Structural model of (D17-m-10-tcga)_1_(T)_2_ complexes (m = 6, 7). Assembly process and a pair of AFM images at different scales for T with D17-m-10-tcga when m = 6 (c) and 7 (d). The measured values of the repeating distances along the red/blue lines on AFM images are indicated on the left to the images. The expected values are calculated from and indicated on the models.


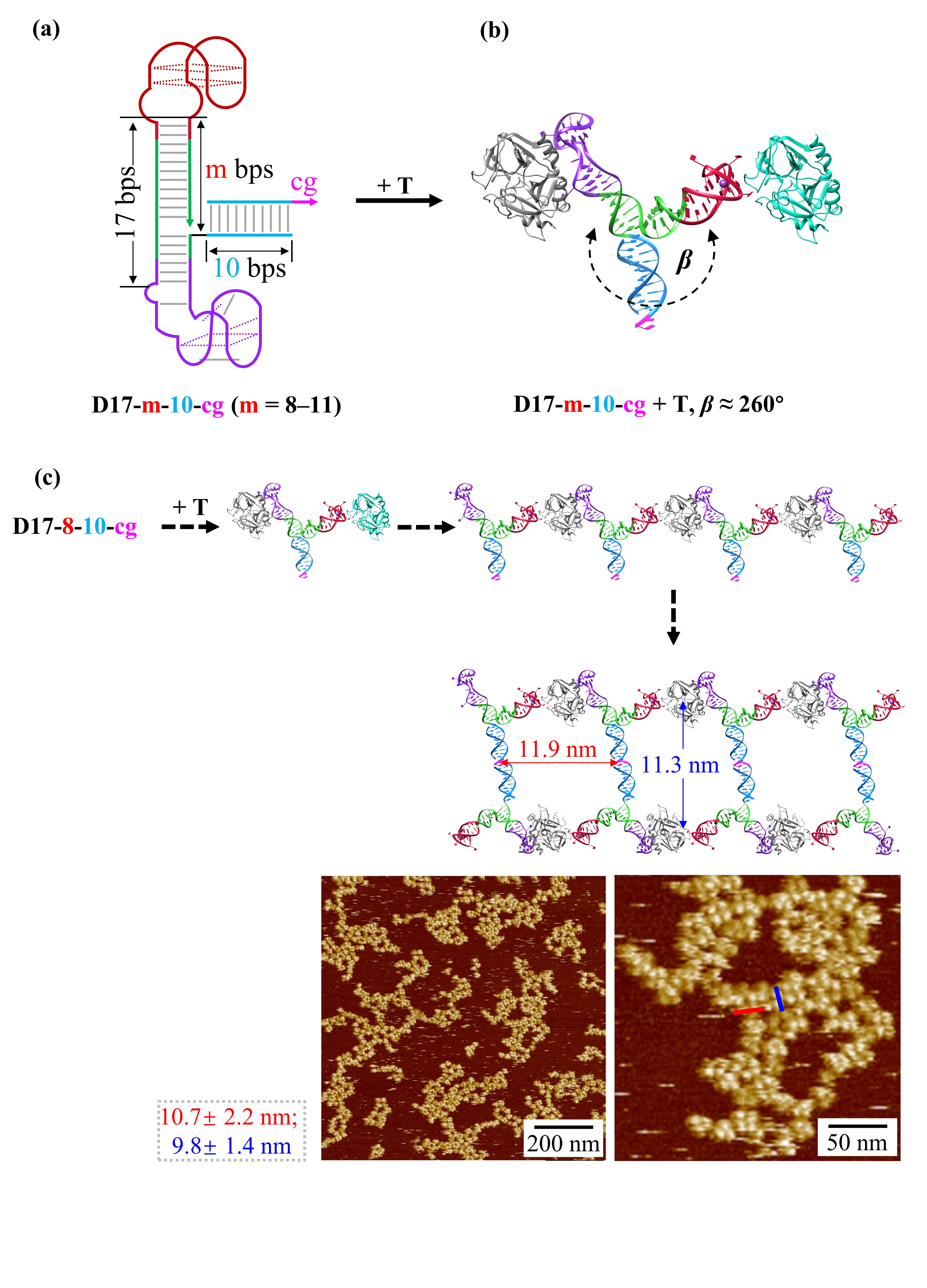

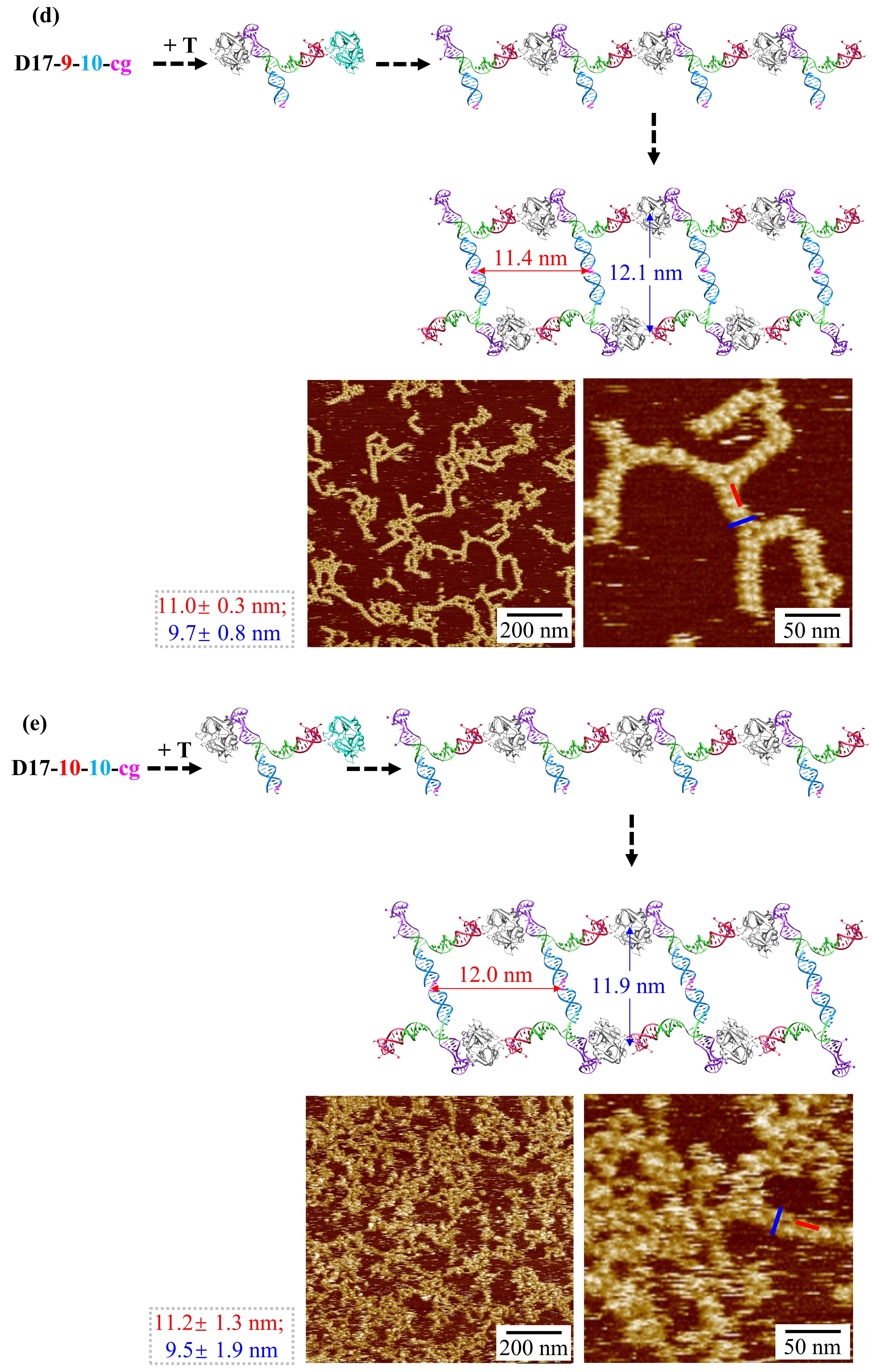

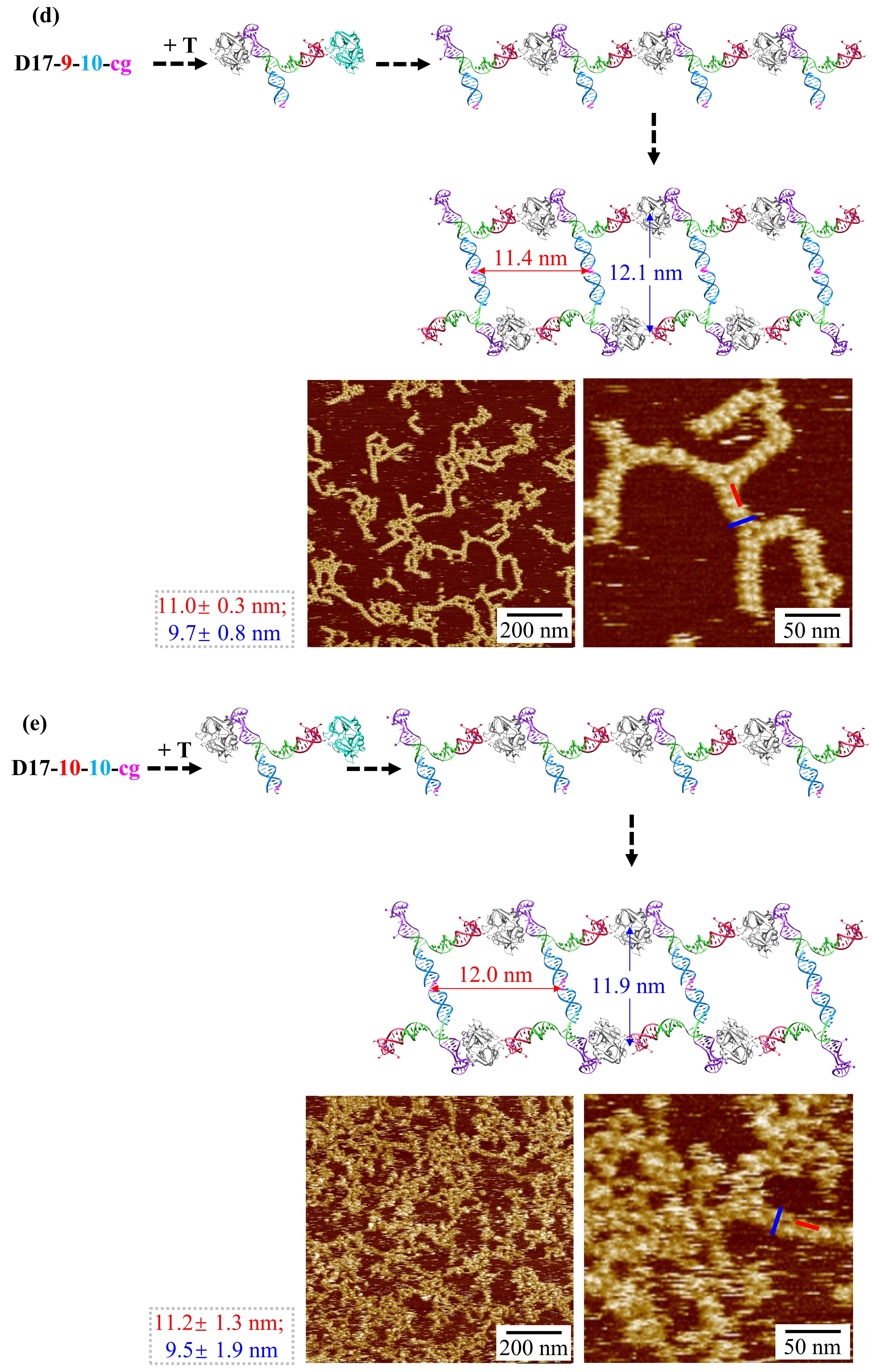


Figure S6**.** Double-connected PDF 1D ladders assembled from D17-m-10-cg (m = 8–10) and T. (a) Scheme of the secondary structure of D17-m-10-cg. (b) Structural model of (D17-m-10-cg)_1_(T)_2_ complexes (m = 8–10). Assembly process and a pair of AFM images at different scales for T with D17-m-10-cg when m = 8 (c), 9 (d), and 10 (e). The measured values of the repeating distances along the red/blue lines on AFM images are indicated on the left to the images. The expected values are calculated from and indicated on the models.


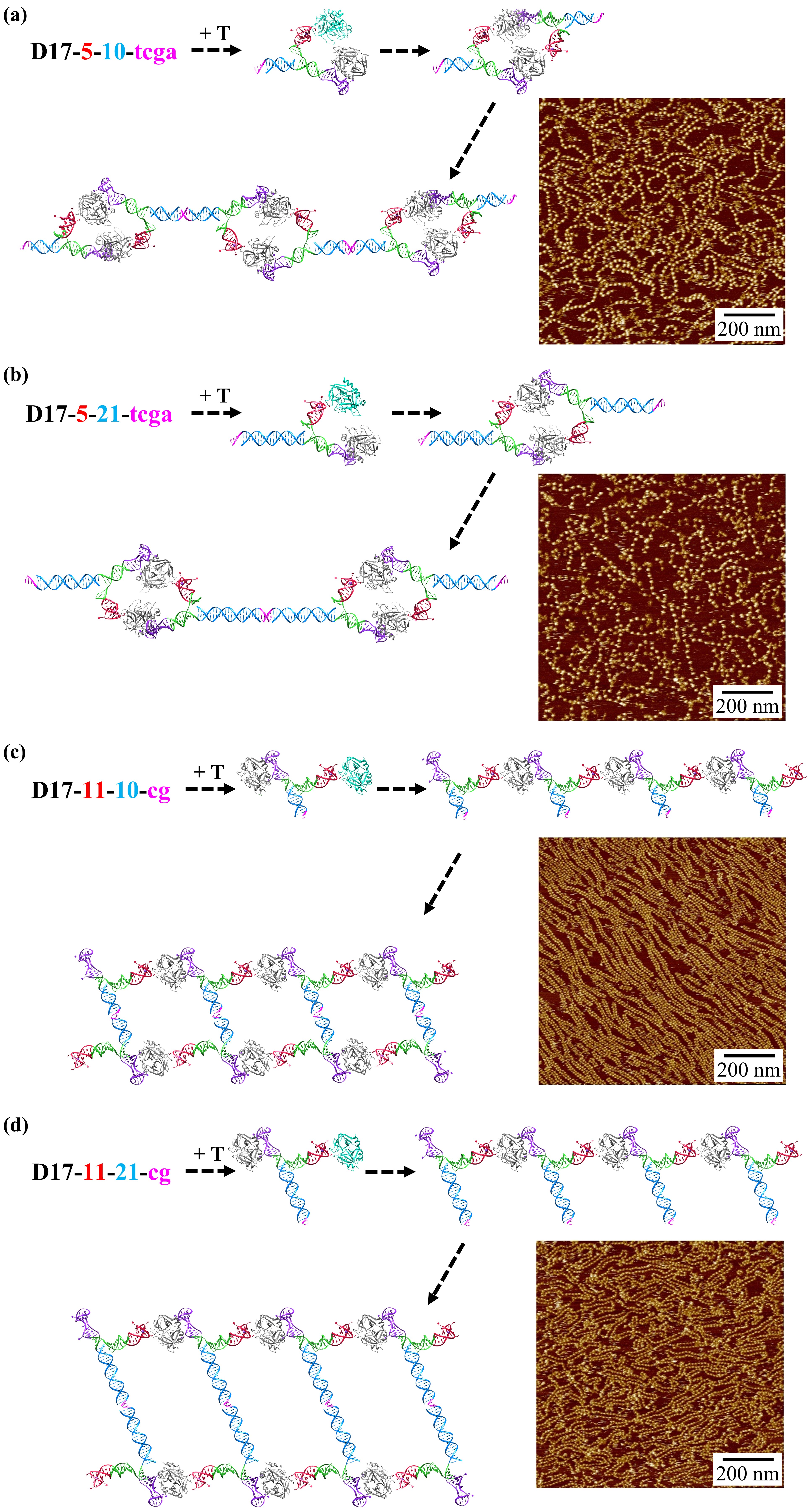

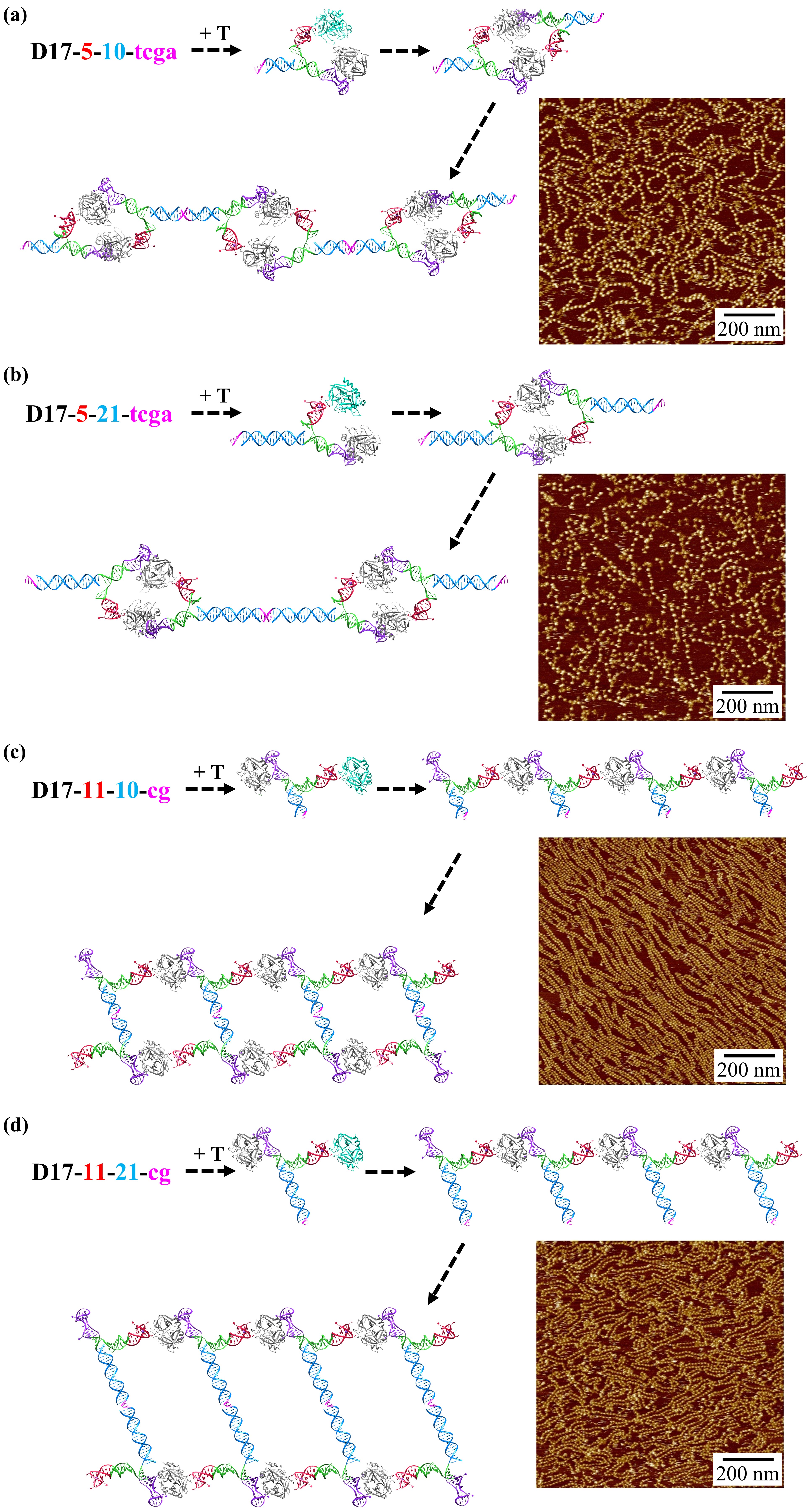


Figure S7**.** Double-connected PDF 1D arrays assembled from D17-m-k-se (m = 5 or 11; k = 10 or 21) and T. Structural models and corresponding AFM images for co-assembly of T and D17-5-10-tcga (a), D17-5-21-tcga (b), D17-11-10-cg (c) and D17-11-21-cg (d).


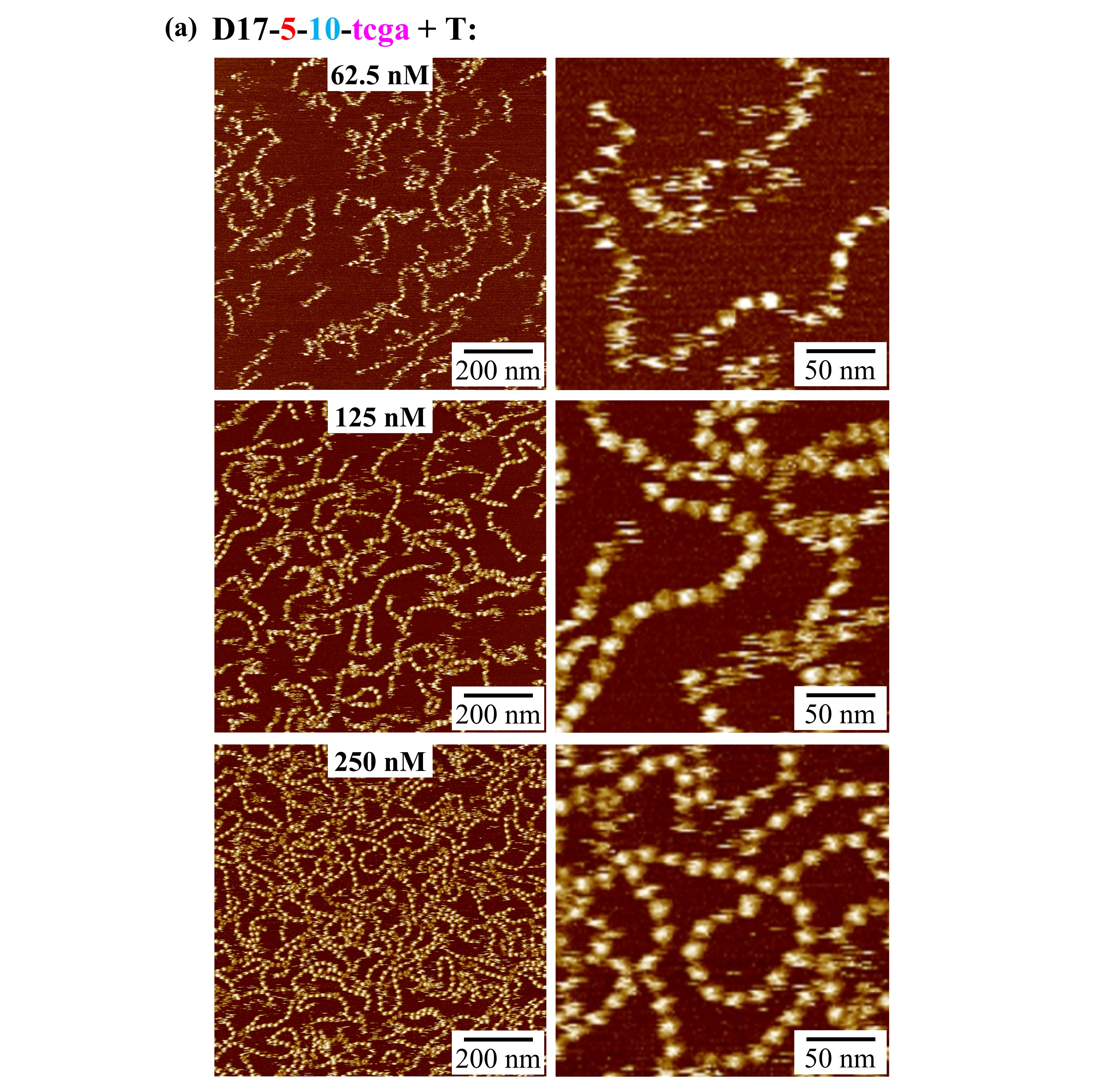


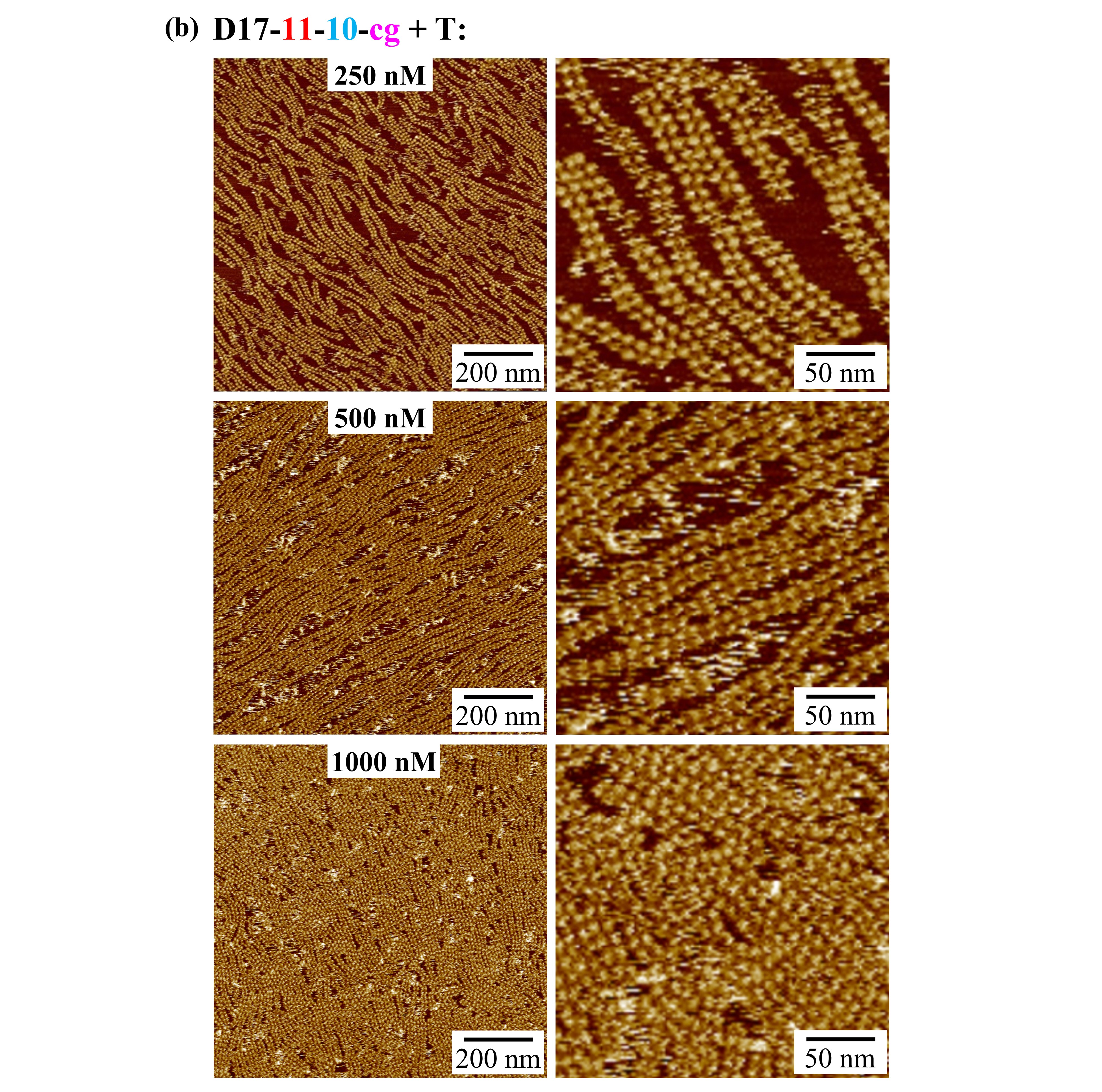


Figure S8**.** AFM images for co-assembly of T and D17-5-10-tcga (a) and D17-11-10-cg (b) at different concentrations.


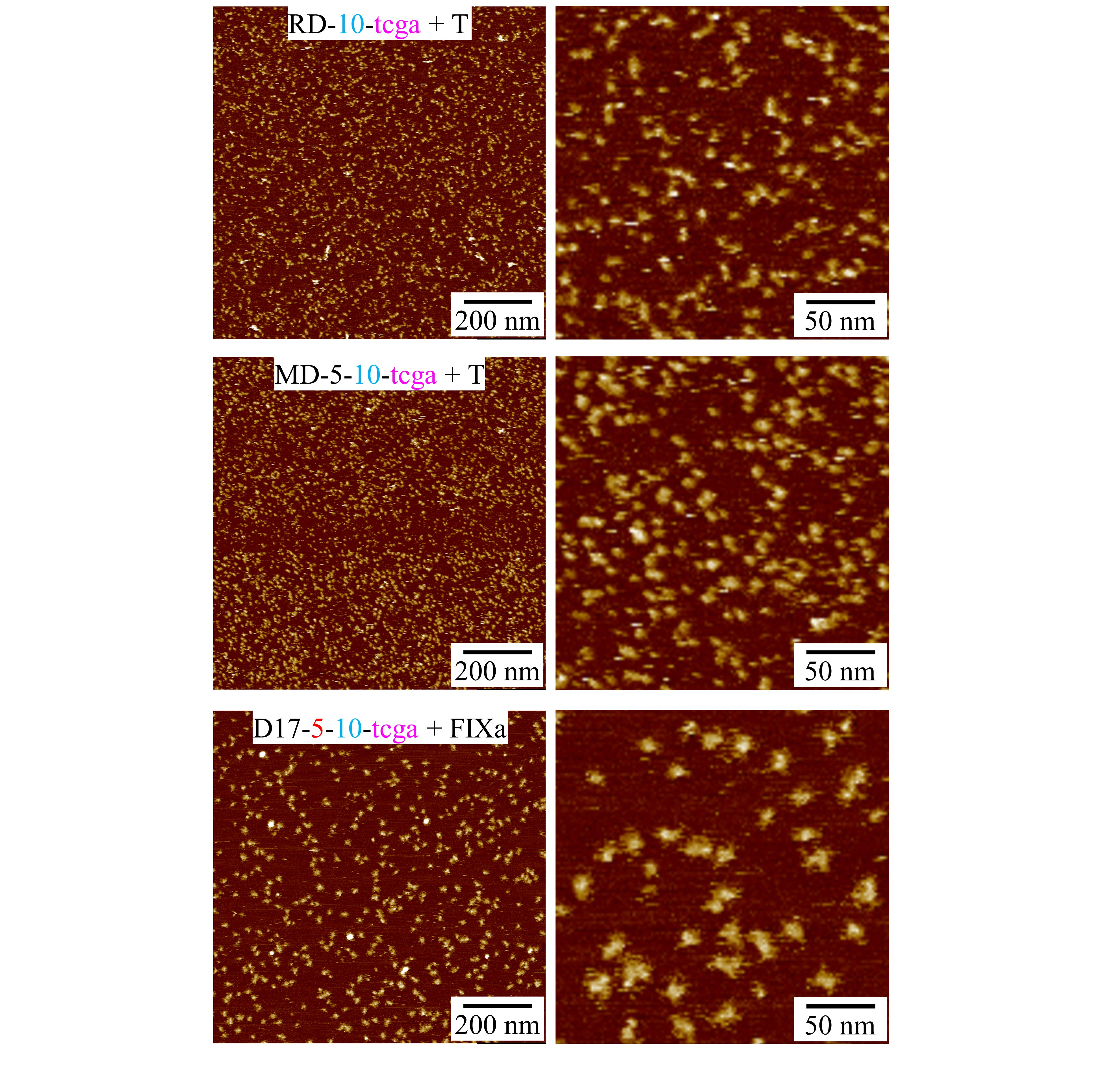


Figure S9. AFM images of assemblies formed by T with random sequence (RD-10-tcga) and mutant sequence (MD-5-10-tcga), and the assembly of D17-5-10-tcga with FIXa.


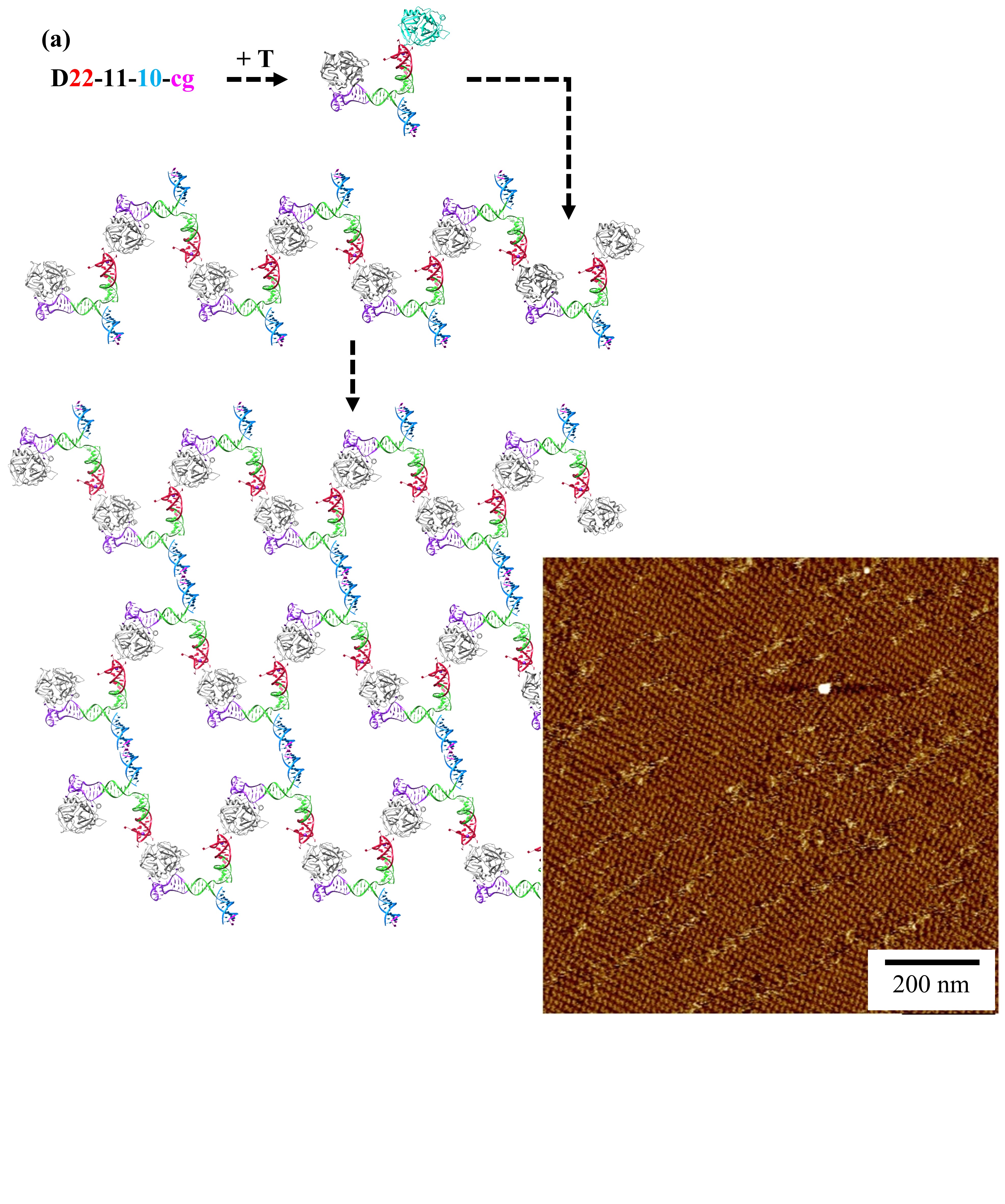

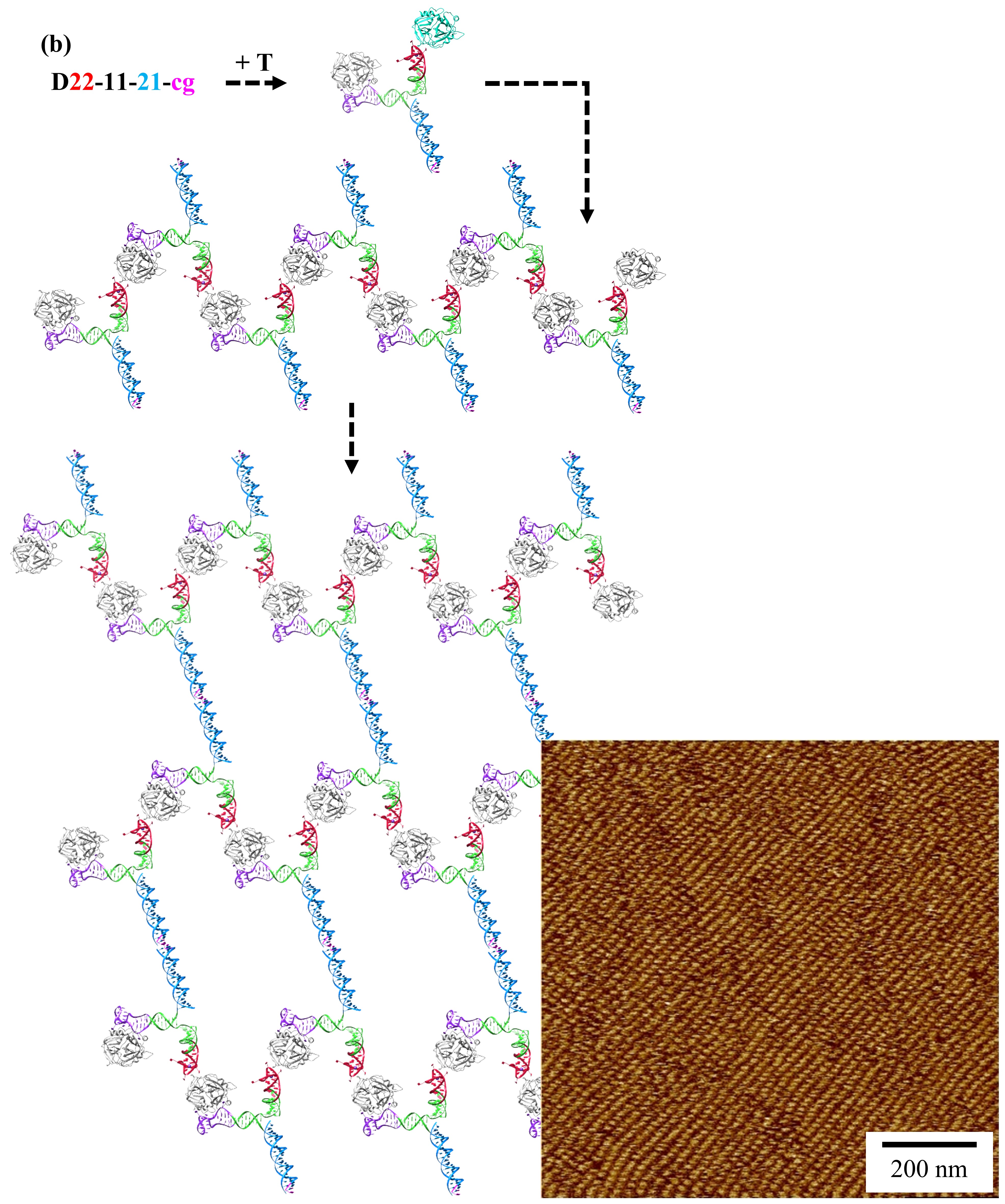


Figure S10**.** 2D arrays assembled from D22-11-k-cg (k = 10 or 21) and T. Structural models and corresponding AFM images for co-assembly of T and D22-11-10-cg (a) and D22-11-21-cg (b).


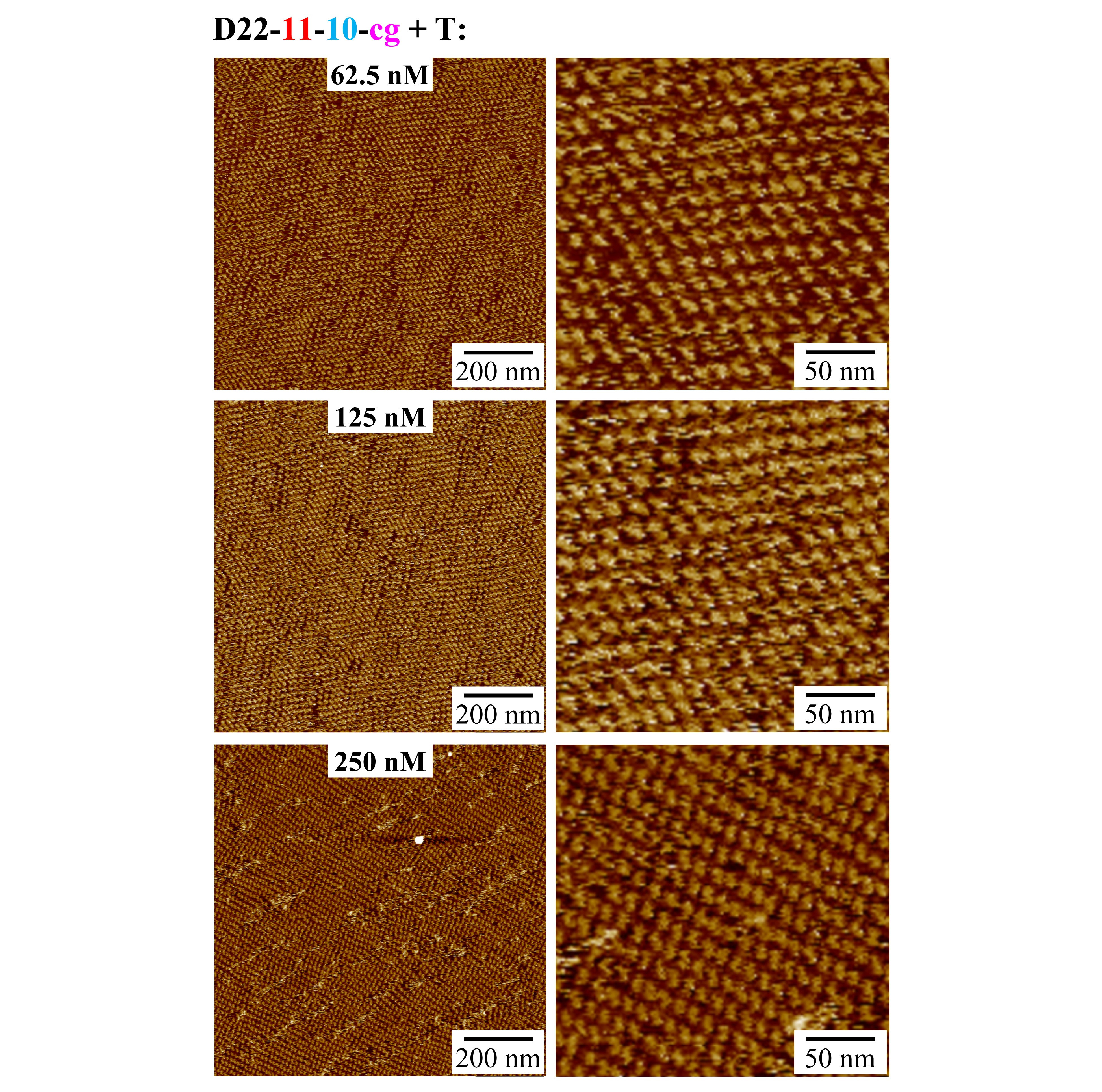


Figure S11**.** AFM images for co-assembly of T and D22-11-10-cg at different concentrations.


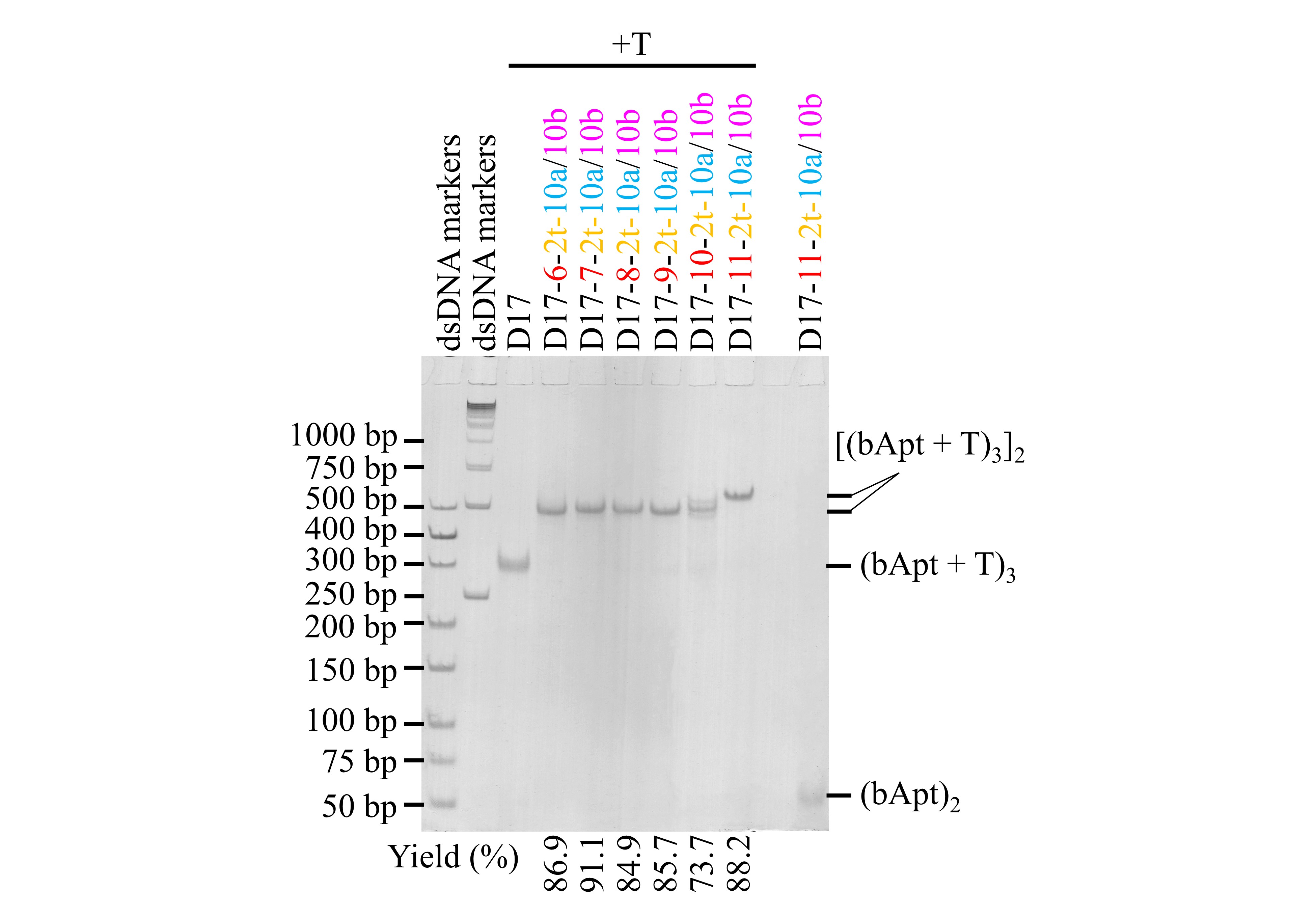


Figure S12**.** nPAGE (4%) characterization of the trigonal PDF prisms assembled from 17-m-2t-10a/10b (m = 6–11) and T. The yields shown below the gel correspond to the prisms in each lane.





Figure S13**.** Cryo-EM data processing workflow for the D17-6-2t-10a/10b-T.





Figure S14**.** Cryo-EM data processing workflow for the D17-11-2t-10a/10b-T.





Figure S15**.** Workflow for constructing the RE31-T-HD22-based trigonal PDF prisms models fitted into the cryo-EM density maps.


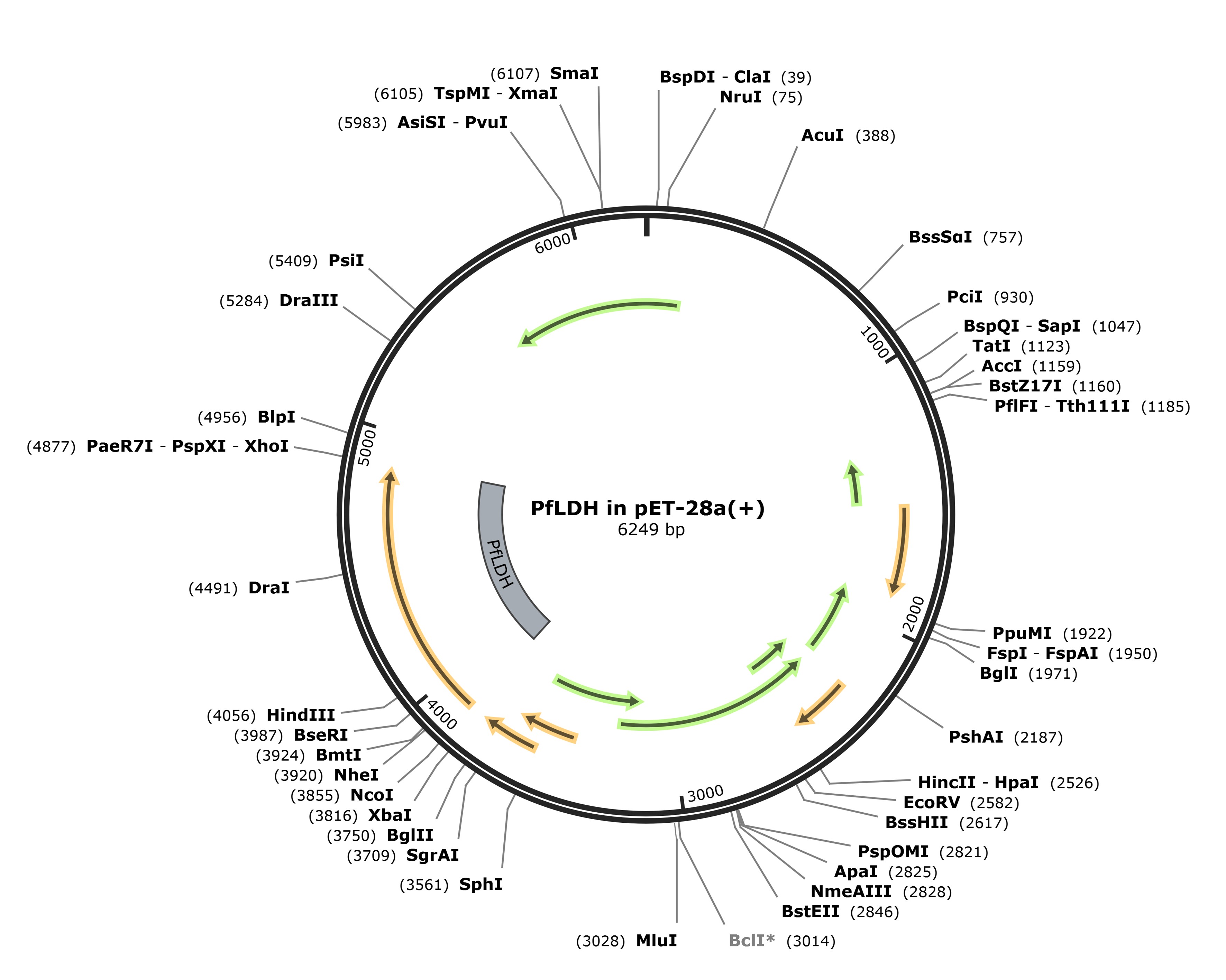


Figure S16**.** Map of the recombinant plasmid encoding pET28a-*Pf*LDH.


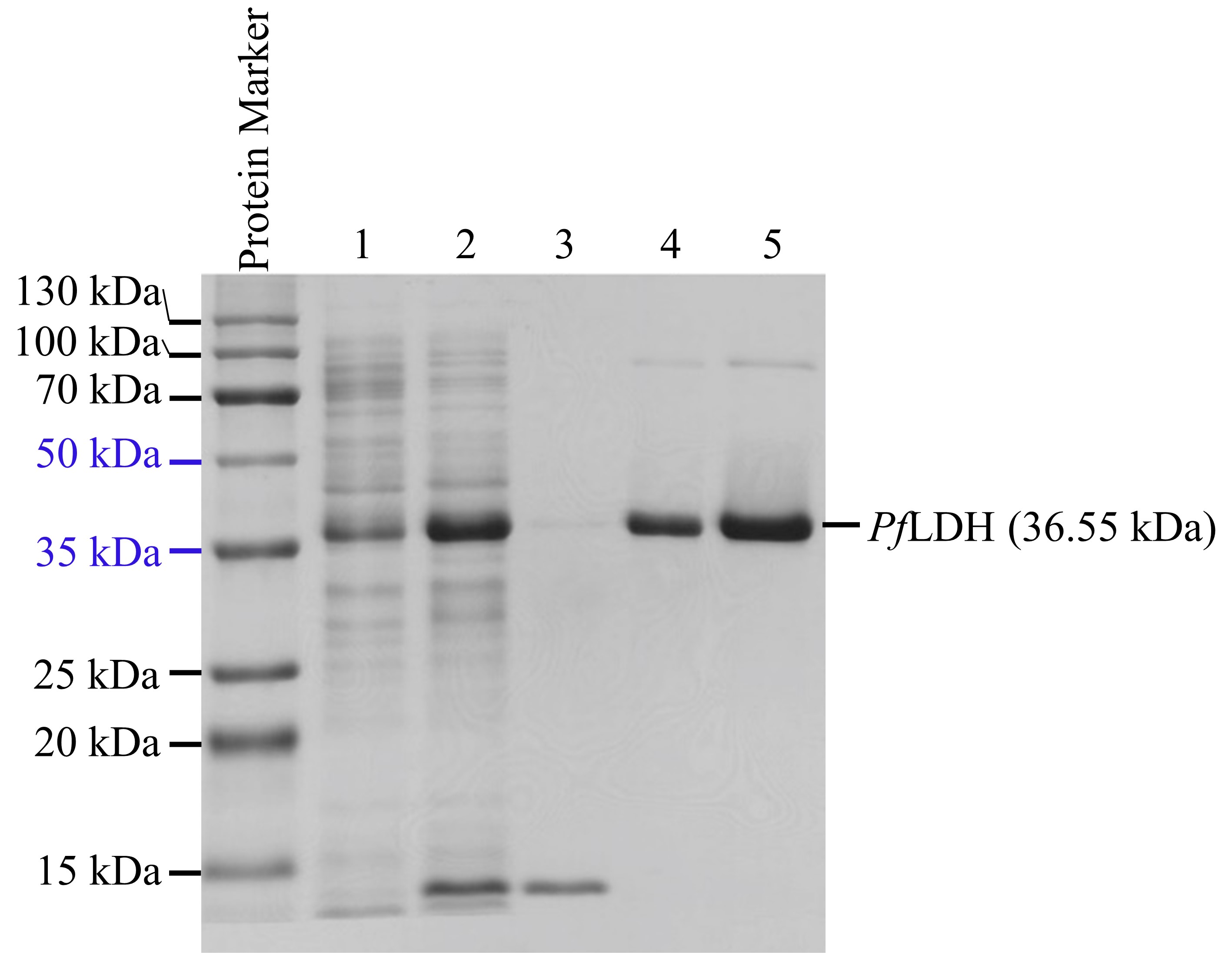


Figure S17**.** SDS-PAGE analysis of the *Pf*LDH protein. Lane 1: whole-cell lysate; Lane 2: supernatant after sonication and centrifugation; Lane 3: flow-through from the Ni-NTA resin; Lanes 4–5: purified and concentrated *Pf*LDH protein solution (10 μg and 20 μg, respectively).


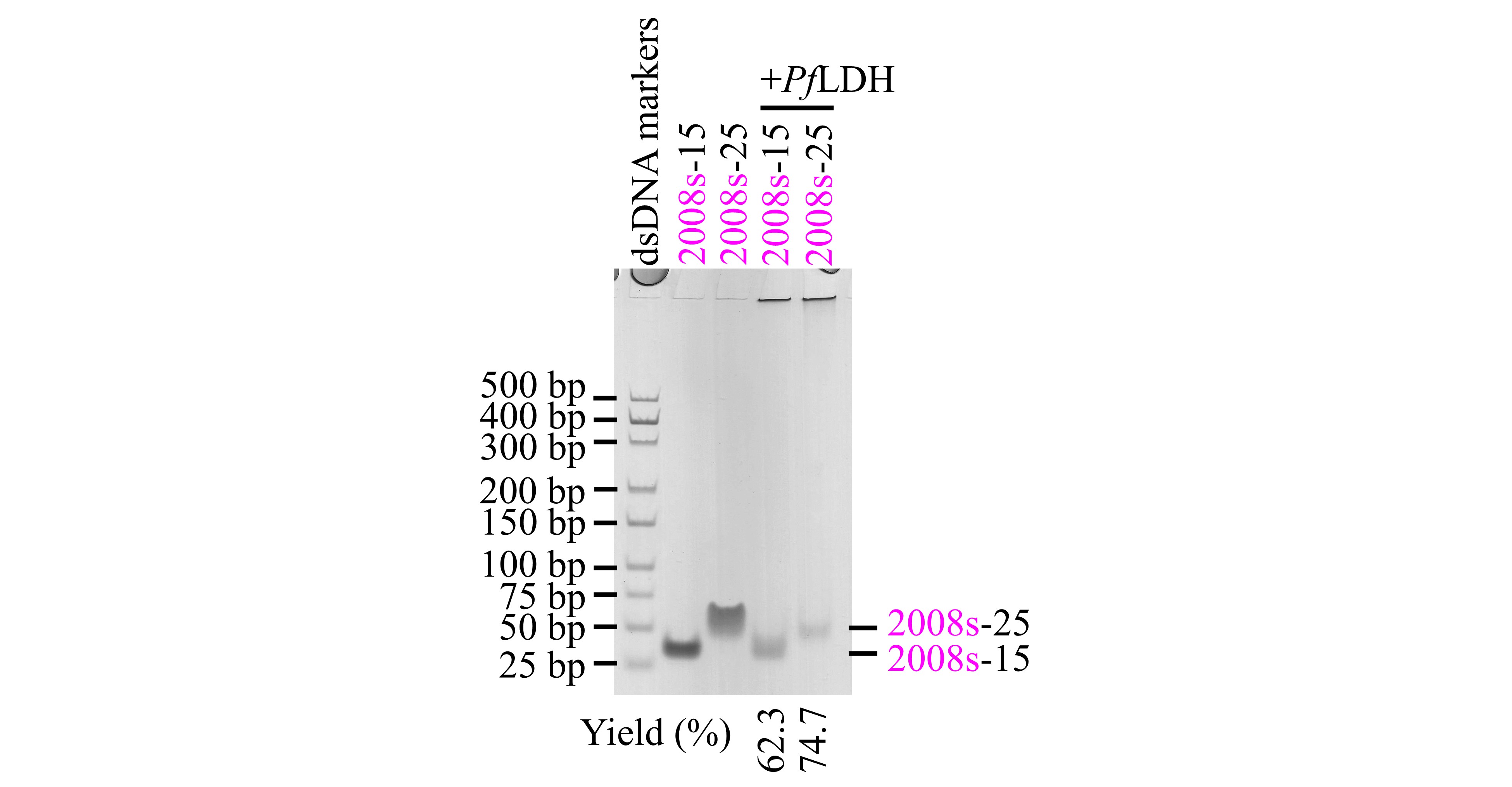


Figure S18**.** nPAGE (4%) characterization of the PDF chains co-assembled by *Pf*LDH and 2008s-x (x = 15 or 25). The yields shown below the gel correspond to the PDF chains in each lane.





Figure S19**.** PDF chains co-assembled by *Pf*LDH and its aptamer 2008s-x. The sequences and secondary structures of 2008s-15 (a) and 2008s-25 (c), along with their co-assembly process with *Pf*LDH. (c) & (d) with long repeating distance. (b) & (d) AFM images of PDF chains. (e) The T-junction cohesion between aptamers.

Table S1**.** Cryo-EM Data collection parameters of D17-6-2t-10a/10b-T.

| Sample | D17-6-2t-10a/10b-T  EMD-68834 |
| --- | --- |
| **Data collection and processing** |  |
| Magnification | 105000x |
| Voltage (kV) | 300 |
| Electron exposure (e^-^/Å^2^) | 50 |
| Defocus range (μm) | -1.0 to -1.6 |
| Pixel size (Å) | 0.8374 |
| Micrographs (No.) | 3,496 |
| Initial particles (No.) | 211,528 |
| Final particles (No.) | 187,786 |
| Map resolution (Å) | 10.63 |

Table S2**.** Cryo-EM Data collection parameters of D17-11-2t-10a/10b-T.

| Sample | D17-11-2t-10a/10b-T  EMD-68835 |
| --- | --- |
| **Data collection and processing** |  |
| Magnification | 29000x |
| Voltage (kV) | 300 |
| Electron exposure (e^-^/Å^2^) | 50 |
| Defocus range (μm) | -1.0 to -1.6 |
| Pixel size (Å) | 0.97 |
| Micrographs (No.) | 2,674 |
| Initial particles (No.) | 50,588 |
| Final particles (No.) | 3,857 |
| Map resolution (Å) | 18.61 |

# References

[1] M. D. Abràmoff, P. Magalhães, S. J. Ram, “Image processing with Image *J*,” *Biophotonics International* **11**, (2004).

[2] K. Zhang, “Gctf: Real-time CTF Determination and Correction,” *Journal of Structural Biology* **193**, (2016): 1–12.

[3] A. Punjani, J. L. Rubinstein, D. J. Fleet, M. A. Brubaker, “cryoSPARC: Algorithms for Rapid Unsupervised Cryo-EM Structure Determination,” *Nature Methods* **14**, (2017): 290–296.

[4] E. F. Pettersen, T. D. Goddard, C. C. Huang, G. S. Couch, D. M. Greenblatt, E. C. Meng, T. E. Ferrin, “UCSF Chimera - A Visualization System for Exploratory Research and Analysis,” *Journal of Computational Chemistry* **25**, (2004): 1605–1612.

[5] H. Zhang, D. H. Zheng, Q. R. Wu, N. Yan, H. Peng, Q. Hu, Y. Peng, Z. F. Yan, Z. Q. Shi, C. L. Bao, M. X. Hu, “CryoPROS: Correcting Misalignment Caused by Preferred Orientation Using AI-generated Auxiliary Particles,” *Nature Communications* **16**, (2025): 4565.
